# Systematic analysis of *DEMETER*-like DNA glycosylase genes shows lineage-specific Smi-miR7972 involved in *SmDML1* regulation in *Salvia miltiorrhiza*

**DOI:** 10.1038/s41598-018-25315-w

**Published:** 2018-05-08

**Authors:** Jiang Li, Caili Li, Shanfa Lu

**Affiliations:** Institute of Medicinal Plant Development, Chinese Academy of Medical Sciences & Peking Union Medical College, No.151 Malianwa North Road, Haidian District, Beijing, 100193 China

## Abstract

DEMETER-like DNA glycosylases (DMLs) initiate the base excision repair-dependent DNA demethylation to regulate a wide range of biological processes in plants. Six putative *SmDML* genes, termed *SmDML1*–*SmDML6*, were identified from the genome of *S*. *miltiorrhiza*, an emerging model plant for Traditional Chinese Medicine (TCM) studies. Integrated analysis of gene structures, sequence features, conserved domains and motifs, phylogenetic analysis and differential expression showed the conservation and divergence of *SmDMLs*. *SmDML1*, *SmDML2* and *SmDML4* were significantly down-regulated by the treatment of 5Aza-dC, a general DNA methylation inhibitor, suggesting involvement of *SmDMLs* in genome DNA methylation change. *SmDML1* was predicted and experimentally validated to be target of Smi-miR7972. Computational analysis of forty whole genome sequences and almost all of RNA-seq data from Lamiids revealed that *MIR7972s* were only distributed in some plants of the three orders, including Lamiales, Solanales and Boraginales, and the number of *MIR7972* genes varied among species. It suggests that *MIR7972* genes underwent expansion and loss during the evolution of some Lamiids species. Phylogenetic analysis of *MIR7972s* showed closer evolutionary relationships between *MIR7972s* in Boraginales and Solanales in comparison with Lamiales. These results provide a valuable resource for elucidating DNA demethylation mechanism in *S*. *miltiorrhiza*.

## Introduction

*Salvia*, widely distributed in the world, is the largest genus in the Labiatae family. It includes about 900 species, of which many have significant economical and medicinal value. *S*. *miltiorrhiza* Bunge is a well-known *Salvia* species widely used in traditional Chinese medicine (TCM) for the treatment of dysmenorrhoea, amenorrhoea and cardiovascular diseases^1–4^. It is also one of the best selling TCM materials with long usage history and the first Chinese herb entering international market. Recently, *S*. *miltiorrhiza* is emerging as a model system for medicinal plant biology^5^. The whole genomes of two *S*. *miltiorrhiza* lines have been decoded^6,7^. A huge amount of RNA-seq-based transcriptome data have been obtained and are available for comparative analysis (https://www.ncbi.nlm.nih.gov/sra). It provides useful information for further elucidating the genetic and epigenetic regulatory mechanisms of *S*. *miltiorrhiza* development and bioactive compound production.

Epigenetic regulation is an important regulatory mechanism affecting many plant cellular processes. It is known that epigenetic phenotypes are caused by changes in a chromosome without alterations in the DNA sequence, including individual or combined changes in DNA methylation and histone modification, as well as the action of chromatin-remodeling factors and noncoding RNAs^8^. DNA methylation is an important epigenetic modification. It is involved in multiple biological processes, such as transposon silencing, genomic imprinting, and X-chromosome inactivation^9–13^. In mammals, DNA methylation predominantly occurs in CG sequence contexts^14^; however, in plants, three sequence contexts, including CG, CHG and CHH (H represents either A, T or G), are major DNA methylation targets^15,16^. DNA methylation levels and patterns in plants are dynamically regulated by DNA methyltransferases and demethylases. DNA METHYLTRANSFERASE 1 (MET1) and CHROMOMETHYLASE 3 (CMT3) maintain symmetric CG^17^ and CHG methylation^18^, respectively. Methylation of asymmetric CHH contexts must be *de novo* created by DOMAINS REARRANGED METHYLTRANSFERASE (DRM) during cell replication^19^. DNA demethylation can be regulated through passive or active processes. Passive demethylation occurs in newly synthesized DNA strand and is caused by dysfunction of DNA methyltransferase, whereas active demethylation is an outcome of replacement of methylated cytosine with non-methylated cytosine under the catalysis of DEMETER-like DNA glyscosylases (DMLs)^20,21^.

The DMLs act as bifunctional glycosylase/AP-lyase, which removes 5-methylcytosine (5 mC) followed by cleaving the abasic site^8^. They contain three essential domains, including a DNA glycosylase domain and two additional conserved domains, termed domain A and domain B^22^. The DNA glycosylase domain has a helix-hairpin-helix (HhH) motif, a glycine/proline-rich region followed by a conserved aspartic acid (GPD), and a [4Fe-4S] cluster motif^22^. The [4Fe-4S] cluster motif consists of four cysteine residues functioning to hold a [4Fe-4S] cluster and is required for 5 mC excision^22^. Amino acids in domain A are required for nonspecific DNA binding and all three domains are necessary and sufficient for 5 mC excision^22^.

DMLs play significant roles in many developmental and biological processes, such as reproduction, seed development, and plant response to biotic and abiotic stresses^20,23–27^. *Arabidopsis* contains four *DML* gene family members, known as *AtDME*, *AtROS1*, *AtDML2* and *AtDML3*, respectively. *AtDME* is necessary for seed viability and is a core regulator of imprinted genes^28^. It is preferentially expressed in the central cell before fertilization, ensuring maternal expression of the imprinted genes *MEA* and *FWA*^29,30^. *AtROS1*, *AtDML2* and *AtDML3* are broadly expressed in vegetative tissues to maintain a proper genome methylation pattern and to regulate relevant genes and transposons^31^. *AtROS1* counteracts RNA-directed DNA Methylation (RdDM) pathway for dynamic transcriptional regulation^32^, whereas *AtDML2* and *AtDML3* are involved in removing improper methylated cytosines and maintaining methylation levels of certain targeted sites to make sure an appropriate distribution of genome methylation^31^. A total of six *DML* genes exist in the rice genome. It includes *OsROS1a*, *OsROS1b*, *OsROS1c*, *OsROS1d*, *OsDML3a* and *OsDML3b*, of which four are *AtROS1* orthologs, whereas the other two are *AtDML3* orthologs^24^. The function of *OsROS1a* is analogous, to certain extent, to that of *AtDME* in both male and female gametophytes^33^. *OsROS1c* is responsible for the demethylation of retrotransposon *Tos17* and plays a critical role in promoting the transposition of *Tos17*^21^. Little is known about the function of other rice *DMLs* and *DMLs* in other plants.

Based on current knowledge of epigenetics, we presume that epigenetic regulation is involved in secondary metabolism and Dao-di herb formation, two important issues in medicinal plant biology. With the long-term goal to test this hypothesis and to elucidate the underlying mechanisms, we analyze and report here the *DML* gene family in *S*. *miltiorrhiza*. Comparative analysis showed that *SmDMLs* were conserved with *Arabidopsis* and rice *DMLs*. They differentially expressed in various *S*. *miltiorrhiza* tissues and were responsive to DNA methylation inhibitor (5-aza-2′-deoxycytidine, 5Aza-dC) treatments. The expression of *SmDML1* is posttranscriptionally regulated by Smi-miR7972. The existence of *MIR7972s* in plants was systematically investigated. *MIR7972* genes were identified in some species belonging to three orders, including Lamiales, Solanales, and Boraginales. The distribution patterns were scattered and the number of gene members was varied among species. The results provide the first hand of information for elucidating the role of epigenetic regulation in medicinal plants.

## Results

### Identification and comparative analysis of *SmDMLs*

Since *S*. *miltiorrhiza* has significant medicinal value and is being developed rapidly as a model system for medicinal plant biology, two research groups sequenced the whole genomes of two different *S*. *miltiorrhiza* lines, one of which is known as 99-3^7^, whereas the name of the other one is unknown^6^. The draft genome assemblies of line 99-3 and the name-unknown line are about 559 and 641 Mb, respectively, although the estimated genome sizes are 615 and 645.78 Mb, respectively^6,7^. It suggests that both of the current *S*. *miltiorrhiza* genome assemblies are incomplete. In order to identify *S*. *miltiorrhiza DEMETER*-like DNA glycosylase gene family (*SmDMLs*), we searched the two genome assemblies using four *Arabidopsis* AtDML proteins as queries. The retrieved genomic DNA sequences were first computationally predicted for gene models on the GENSCAN web server^34^ and then manually examined and corrected by comparison with *DML* genes identified from other plants (www.ncbi.nlm.nih.gov/blast/) and by alignment with RNA-seq data of *S*. *miltiorrhiza* transcriptome (http://www.ncbi.nlm.nih.gov/sra). After correcting various genomic DNA sequence errors, we obtained six full-length *SmDML* genes from the genome assembly of the name-unknown *S*. *miltiorrhiza* line and four full-length *SmDML* genes and several *SmDML* gene fragments from the genome assembly of line 99-3. Amino acid sequence alignment showed that each of the four SmDML proteins from line 99-3 was identical to a SmDML protein from the name-unknown *S*. *miltiorrhiza* line. Sequence comparison of line 99-3 *SmDML* gene fragments, transcriptome of line 99-3 and the other two *SmDML* genes from the name-unknown *S*. *miltiorrhiza* line showed that line 99-3 *SmDML* gene fragments encoded partial SmDML proteins identical to the other two SmDML proteins from the name-unknown *S*. *miltiorrhiza* line. Taken together, we conclude that the *SmDML* gene family includes six members, which are designated as *SmDML1*–*SmDML6*, respectively (Table 1). The number of *SmDMLs* is more than *AtDMLs*, whereas it is same as *OsDMLs*^24^.

**Table 1:** Sequence features and intron numbers of *SmDMLs*, *AtDMLs* and *OsDMLs*.

Gene name	Gene model	Gene length	No. of intron	ORF (bp)	Protein (aa)	MW (kDa)	p*I*
*SmDML1*	MG602215	9194	18	5865	1954	217.9	6.90
*SmDML2*	MG602216	8800	19	5598	1865	207.2	6.97
*SmDML3*	MG602217	11369	19	6093	2030	226.5	8.20
*SmDML4*	MG602218	4976	19	2163	720	80.5	9.27
*SmDML5*	MG602219	6859	20	3165	1054	118.4	6.76
*SmDML6*	MG602220	9820	20	3237	1078	121.8	7.37
*AtDME*	AT5G04560.2	8306	18	5964	1987	221.1	7.61
*AtROS1*	AT2G36490.1	6014	18	4182	1393	156.5	7.38
*AtDML2*	AT3G10010.1	6197	19	3999	1332	151.5	8.62
*AtDML3*	AT4G34060.1	5251	19	3135	1044	120.3	9.63
*OsROS1a*	LOC_Os01g11900.1	11143	16	5859	1952	215.9	6.77
*OsROS1b*	LOC_Os02g29230.1	8100	14	4911	1636	182.6	6.53
*OsROS1c*	LOC_Os05g37350.1	12675	19	5544	1847	205.9	6.61
*OsROS1d*	LOC_Os05g37410.1	10616	20	5490	1829	204.3	6.53
*OsDML3a*	LOC_Os02g29380.1	8438	16	3624	1207	134.4	6.14
*OsDML3b*	LOC_Os04g28860.1	5860	12	2883	960	108.4	8.94

**Figure 1:**
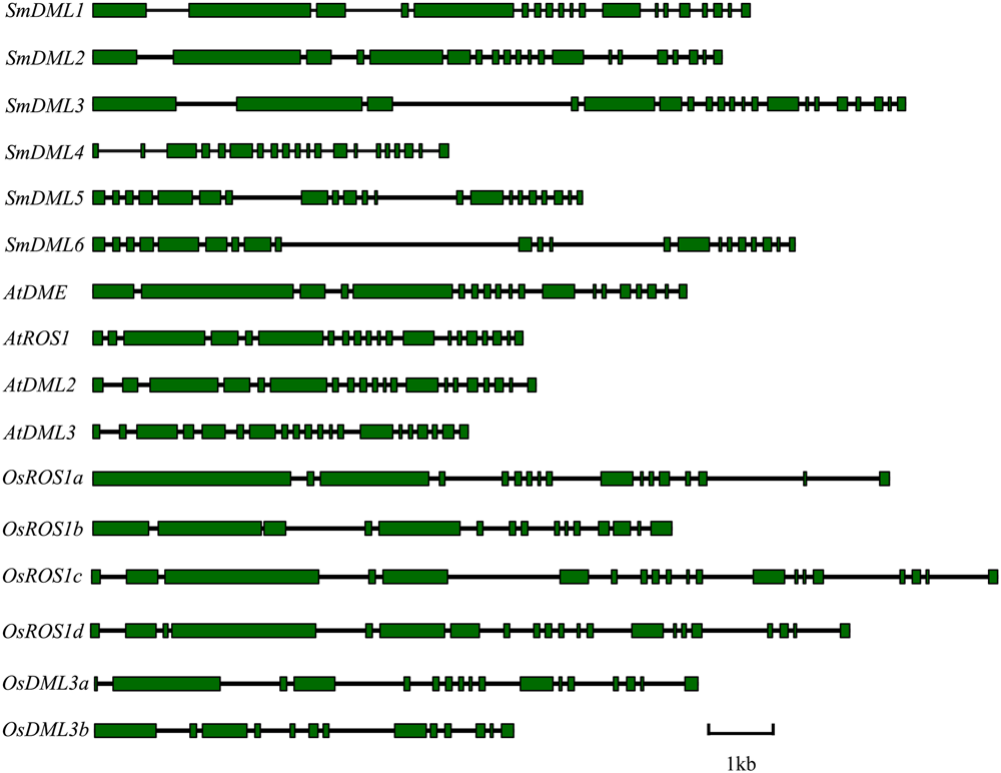
Gene structures of *DMLs* in *S. miltiorrhiza*, *Arabidopsis* and rice. Exons are presented by filled green boxes. Introns are presented by lines.

*SmDML* genes contain 18–20 introns (Table 1, Fig. 1). Although similarities of gene structures exist among the six *SmDMLs*, it is particularly high among *SmDML1*, *SmDML2* and *SmDML3* and between *SmDML5* and *SmDML6*. In addition, the structures of *SmDML1*, *SmDML2* and *SmDML3* are highly similar to four *AtDMLs*, *OsROS1a*–*OsROS1d* and *OsDML3a* (Fig. 1). The deduced SmDML proteins have amino acid number varying from 720 (SmDML4) to 2030 (SmDML3), isoelectric point (p*I*) from 6.76 (SmDML5) to 9.27 (SmDML4), and molecular weight (Mw) from 80.5 kDa (SmDML4) to 226.5 (SmDML3) (Table 1). Wide ranges of gene length, p*I* and Mw also exist among *AtDMLs* and *OsDMLs* (Table 1). It suggests the conservation and divergence of sequence features of plant *DMLs*.

### Conserved domains and motifs of DMLs

**Figure 2:**
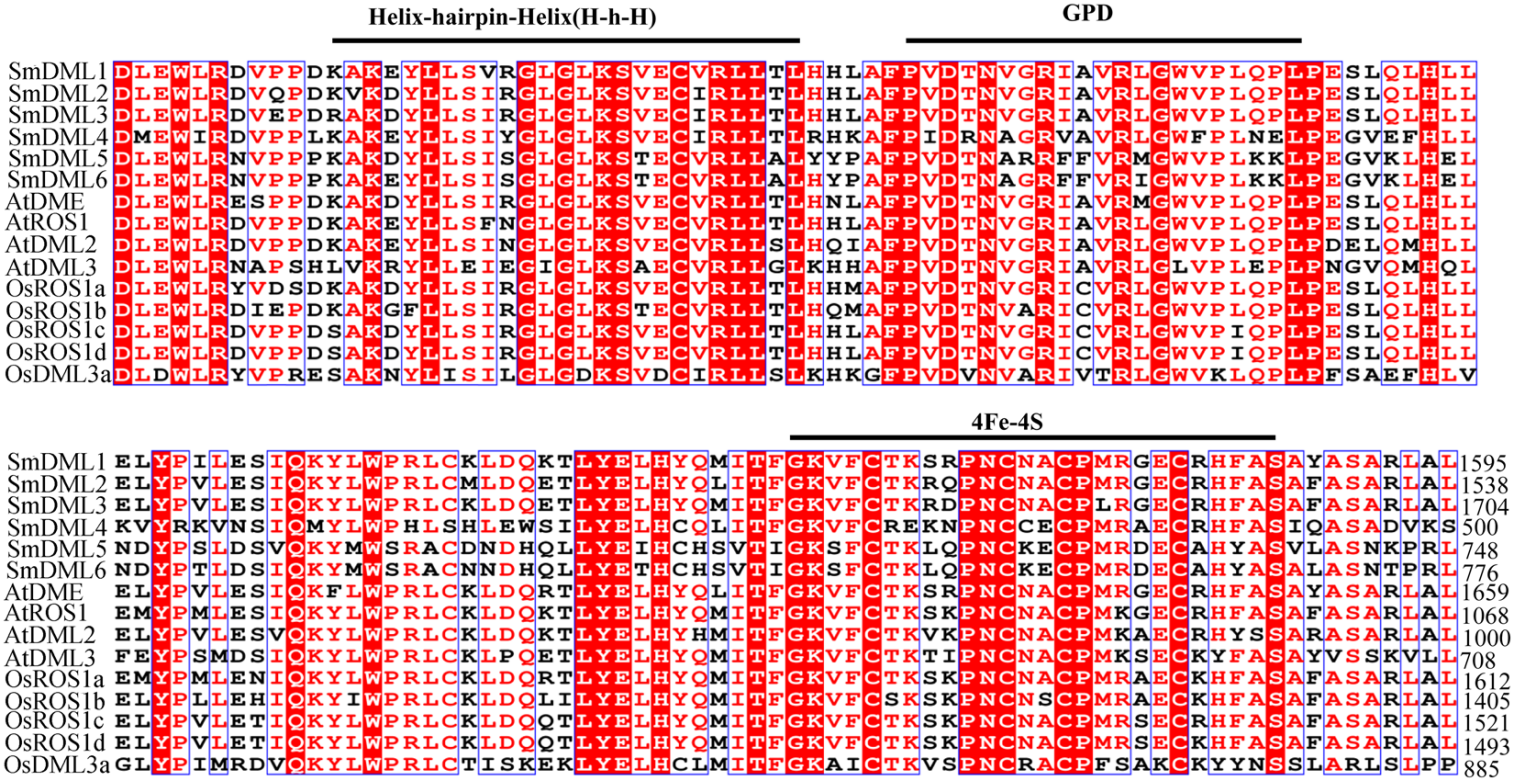
Amino acid sequence alignment of the conserved DNA glycosylase domain of DML proteins in *S*. *miltiorrhiza*, *Arabidopsis* and rice. Numbers show the position of amino acids. Identical amino acids are highlighted in red. Similar amino acid residues are showed in red.

It has been shown that DML proteins contain a DNA glycosylase domain and two additional conserved domains A and B^22^. Multiple sequence alignment of deduced amino acid sequences showed that all of the six SmDMLs also contain the three domains (Fig. 2, see Supplementary Figs S1–3). The DNA glycosylase domain is highly conserved among 15 analyzed DMLs, including six SmDMLs, four AtDMLs and five OsDMLs, and contains HhH, GPD and [4Fe-4S] cluster motifs (Fig. 2). Compared with other DMLs analyzed, OsDML3b has less conserved DNA glycosylase domain. Differing from the DNA glycosylase domain, the sequences of domains A and B are highly conserved among all 16*S*. *miltiorrhiza*, *Arabidopsis* and rice DMLs (see Supplementary Figs S2,3). In addition to the amino acid sequence, the location of domains is conserved among all DMLs from *S*. *miltiorrhiza*, *Arabidopsis* and rice (see Supplementary Fig. S1). These results suggest that the majority of domains in DMLs from different plant species are deeply conserved. It is consistent with the critical function of these domains in 5 mC excision.

**Figure 3:**
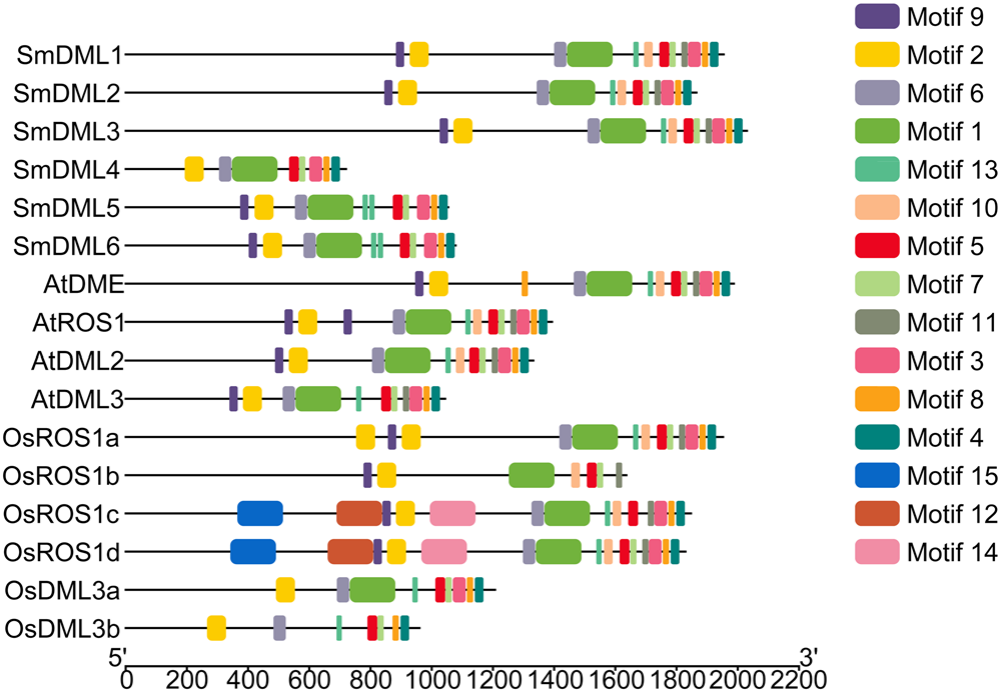
Distribution of conserved motifs of DML proteins from *S*. *miltiorrhiza*, *Arabidopsis* and rice.

In order to further determine the conservation and divergence of plant DMLs, we searched conserved motifs in SmDMLs, AtDMLs and OsDMLs using the MEME suite (http://meme.sdsc.edu/meme/meme.html). A total of 15 conserved motifs were identified (Fig. 3). The length of motifs ranges from 18 to 150 amino acids (Table 2). The number of motifs in each DML varies between 7 and 15. Motif 1 is actually the DNA glycosylase domain. It exists in 15 of the 16 DMLs analyzed (Fig. 3). No motif 1 was detected in OsDML3b. It is consistent with the fact that the DNA glycosylase domain is less conserved in OsDML3b compared with that in other DMLs analyzed. Motifs 2 and 9 are located in domain A. Motif 2 exists in all 16 DMLs analyzed, whereas motif 9 was found in 13 of the 16 DMLs. No motif 9 was detected in SmDML4, OsDML3a and OsDML3b. It suggests that motif 2 is more conserved than motif 9, although both of them are located in the conserved domain A. Motifs 3, 4, 5, 7, 8 and 11 are located in domain B. Among them, motifs 3, 4, 5, 7 and 8 existing in 14 or 15 DMLs show relatively high conservation, whereas motif 11, which was detected in 11 of 16 DMLs, is the least conserved. In addition to the nine motifs found in conserved domains, other six, including motifs 6, 10, 12, 13, 14 and 15, are located in less conserved regions. Among them, motifs 6, 10 and 13 are highly conserved, whereas motifs 12, 14 and 15 are specific to OsROS1c and OsROS1d. Motifs commonly existing in DMLs are probably associated with the conserved functions of DMLs, but those specific to a few DMLs seem to be related to gene-specific functions.

**Table 2:** Consensus sequences of 15 motifs identified in SmDMLs, AtDMLs and OsDMLs.

Motif	Length (aa)	Consensus sequence
1	150	FLNRLVKDHGSIDLEWLRDVPPDKAKDYLLSIRGLGLKSVECVRLLTLHHLAFPVDTNVGRIAVRLGWVPLQPLPESLQLHLLELYPVLESIQKYLWPRL
2	63	ERRVFRGRADSFIARMHLVQGDRRFSPWKGSVVDSVVGVFLTQNVSDHLSSSAFMSLAAKFPV
3	43	RGTILIPCRTAMRGSFPLNGTYFQVNEVFADHESSLNPIDVPR
4	29	QYCFWRGFVCVRGFDRKTRAPRPLVARLH
5	33	ASIPTPKLKNVSRLRTEHQVYELPDSHPLLEGF
6	41	ERTEDTMDSVDWEAVRCADVKEIADTIKERGMNNMLAERIK
7	21	DKREPDDPCPYLLAIWTPGET
8	21	WNLPRRTVYFGTSVPSIFKGL
9	28	VKKKKPRPKVDLDPETTRVWNLLMGKDA
10	29	EAFYEDPDEIPTIKLNMEEFTQNLKSYIQ
11	21	LCSNETCFSCNSIREAQSQTV
12	150	YIKFMTKKRSQKARLNSPNSIQPNIDQKNRFSSETIFSGGFNGLKRSEETFQKTLPQIPDDKRINLDIHCEVPVENSPNTSTPPYMDYLQGVTSKFRYFD
13	18	NCEPIIEEPASPEPEIEE
14	150	MACEKIHMEPKGNATVNELTKGENYSLHCKEPAGSLCDHETEIDHKAKSISDFSAVELTACMKNLHATQFQKEISLSQSVVTSESILQPGLPLSSGMDHA
15	150	HRPKVIREDRPAKKQMATTSEEKPLNQKPKRKYVWKNRNPSSLEKCAEPFSDHSISRESRTTVRSSIASVRRRLQFEFGEHGVQRDQSSRTNSWYRNQEK

### Phylogenetic analysis of DML proteins

**Figure 4:**
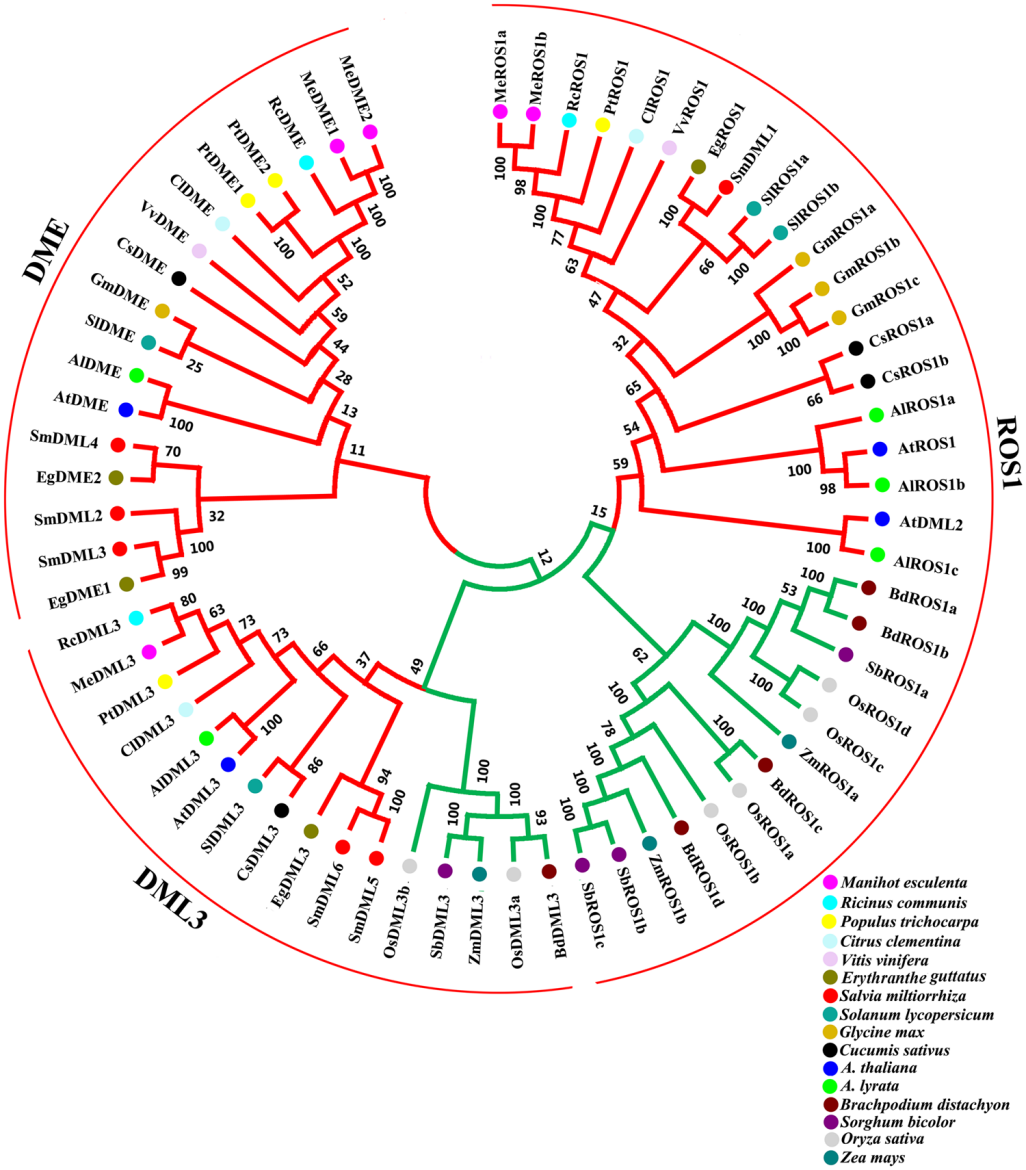
Phylogenetic analysis of DML proteins from 16 plant species. Monocot and dicot are colored green and red, respectively. The bootstrap values are shown.

In order to determine the evolutionary relationship among DMLs, an unrooted neighbor-joining tree was constructed using 66 full length protein sequences from 16 plant species. DML proteins were clustered into three orthology groups, including the DME group, the ROS1 group, and the DML3 group (Fig. 4). The ROS1 group is the largest group. It contains 33 DML proteins. Based on the phylogenetic tree constructed, the ROS1 group may be further divided into the monocot subgroup and the dicot subgroup. Similar to the ROS1 group, the DML3 group may also be divided into the monocot subgroup and the dicot subgroup. DME group is only restricted to dicots. It indicates that DME may be phylogenetically monophyletic in dicots. These results are consistent with previous phylogenetic tree constructed using conserved DNA glycosylase domains of DMLs from flowering plants^24^. It suggests the conservation and divergence of DML proteins in monocots and dicots.

All of SmDMLs showed close phylogenetic relationships with DMLs from monkey flower (*Erythranthe guttatus*) (Fig. 4). It is consistent with the fact that both *S*. *miltiorrhiza* and *E*. *guttatus* are members of Lamiales. Based on gene structures, conserved motifs and phylogenetic relationships, SmDML2 and SmDML5 are closely related with SmDML3 and SmDML6, respectively. SmDML2 and SmDML3 share 62.88% protein sequence identity and 66.16% cDNA similarity. Protein sequence identity and cDNA similarity between SmDML5 and SmDML6 are 89.58% and 93.17%, respectively. It implies duplication events occurred during *SmDML*s evolution. To test the phylogenetic selection pressure on these genes after duplication, we analyzed the substitution rate ratios of nonsynonymous (Ka) versus synonymous (Ks) mutations (Ka/Ks)^35^. Generally, Ka/Ks = 1 suggests that the genes are pseudogenes with neutral selection, whereas less than 1 implies purifying or stabilizing selection^35^. The calculated Ka/Ks values between *SmDML2* and *SmDML3* and between *SmDML5* and *SmDML6* are 0.3833 and 0.9799, respectively. It indicated that *SmDML2* and *SmDML3* paralogous pairs experienced strong purifying selection, whereas *SmDML5* and *SmDML6* paralogous pairs could be pseudogenes with neutral selection. These results suggested that specific *SmDMLs* experienced distinct phylogenetic selection pressure.

### Differential expression of *SmDMLs* in *S*. *miltiorrhiza*

**Figure 5:**
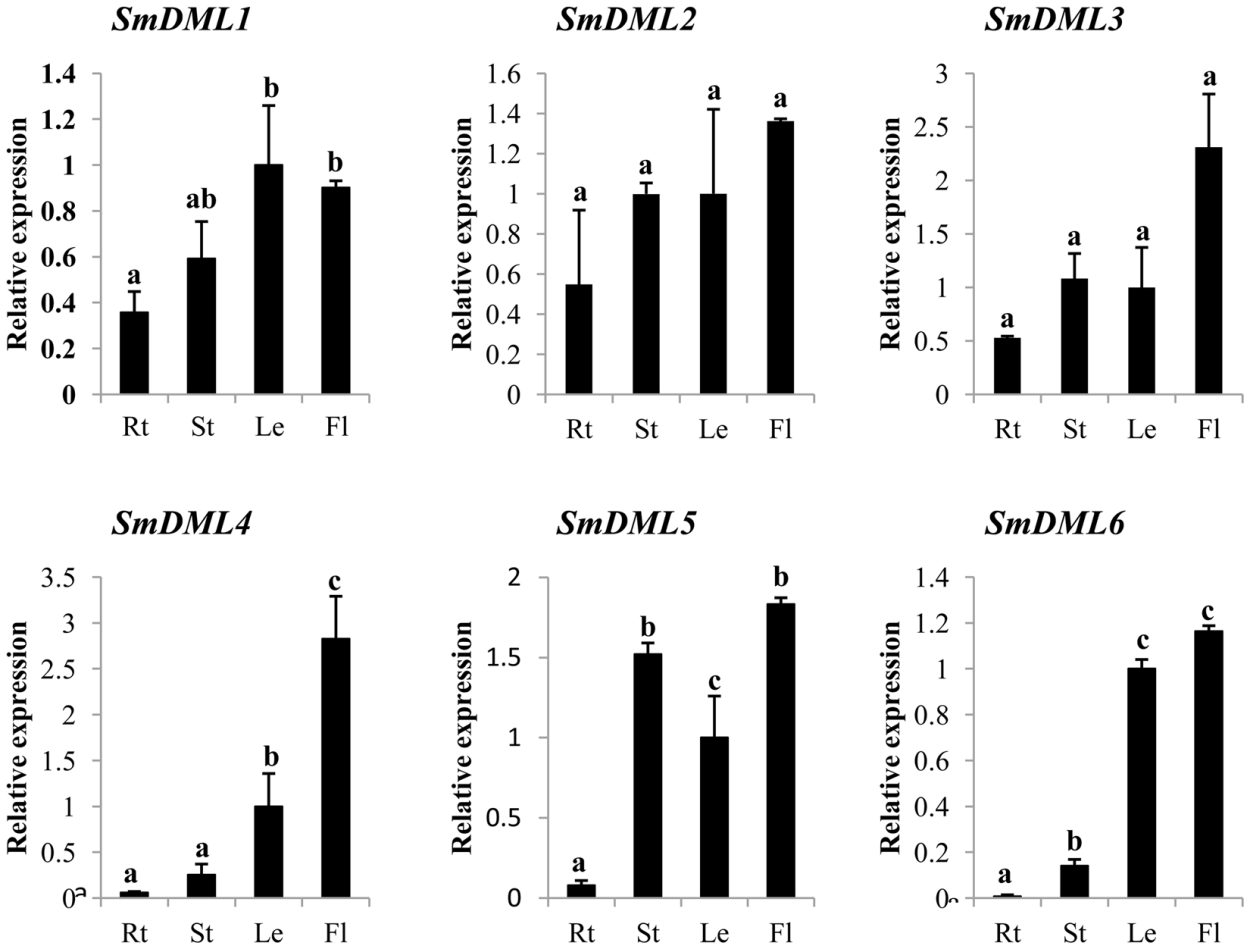
Expression patterns of *SmDMLs* in roots (Rt), stems (St), leaves (Le) and flowers (Fl) of *S*. *miltiorrhiza*. The expression levels were analyzed using the quantitative RT-PCR method. Fold changes of *SmDML* expression are shown. Expression level in leaves was arbitrarily set to 1 and the levels in other organs were given relative to this. One-way ANOVA was calculated using IBM SPSS 20 software. *P* < 0.05 was considered statistically significant and was represented by different letters. Error bars was indicated by the standard deviations of three biological replicates.

As the main participants of DNA demethylation, DMLs play important roles in plant growth and development^36,37^. To preliminarily explore the biological function of *SmDMLs*, the expression of six *SmDMLs* in flowers, leaves, roots and stems of 2-year-old, field nursery-grown *S*. *miltiorrhiza* plants was analyzed using quantitative RT-PCR technology. Although the transcripts of all six *SmDMLs* were detected in the tissues analyzed, significant differential expression patterns were observed (Fig. 5). *SmDML1* showed the highest expression in leaves and flowers. Its expression in roots was the lowest among the four tissues analyzed. The expression patterns of *SmDML1* are different from its *Arabidopsis* counterpart, *AtROS1*, which showed high expression in stems and roots and low in flowers^31^. *SmDML2* and *SmDML3* are paralogs closely related (Fig. 4). Consistently, they showed similar expression patterns with the highest in flowers, followed by stems, leaves and roots. *SmDML4* was predominantly expressed in flowers. Although *SmDML5* and *SmDML6* are probably pseudogenes based on Ka/Ks, they were expressed. *SmDML5* showed the highest expression in flowers and stems, followed by leaves, and the lowest in roots, whereas *SmDML6* was expressed mainly in flowers and leaves (Fig. 5). Expressed pseudogenes have been reported in other plants, such as rice^35^ and barley^38^.

To further analyze the expression patterns of *SmDMLs*, we analyzed RNA-seq data generated for periderm, phloem and xylem of *S*. *miltiorrhiza* roots. *SmDML1* exhibited the highest expression in xylem, followed by phloem and periderm. The expression of *SmDML2* and *SmDML3* paralogs showed similar patterns with the highest in phloem, followed by xylem and periderm. However, the differential expression for each of them in the three tissues of *S*. *miltiorrhiza* roots was not significant as analyzed using TopHat2.0.12 and Cufflinks2.2.1^39^ (Table S3). The FPKM (Fragments Per Kilobase of transcript per Million mapped reads) values of *SmDML4–SmDML6* were very low, which are consistent with the low expression levels in roots analyzed using the quantitative RT-PCR technology (Fig. 5).

### Expression patterns of *SmDML* genes in response to 5Aza-dC

**Figure 6:**
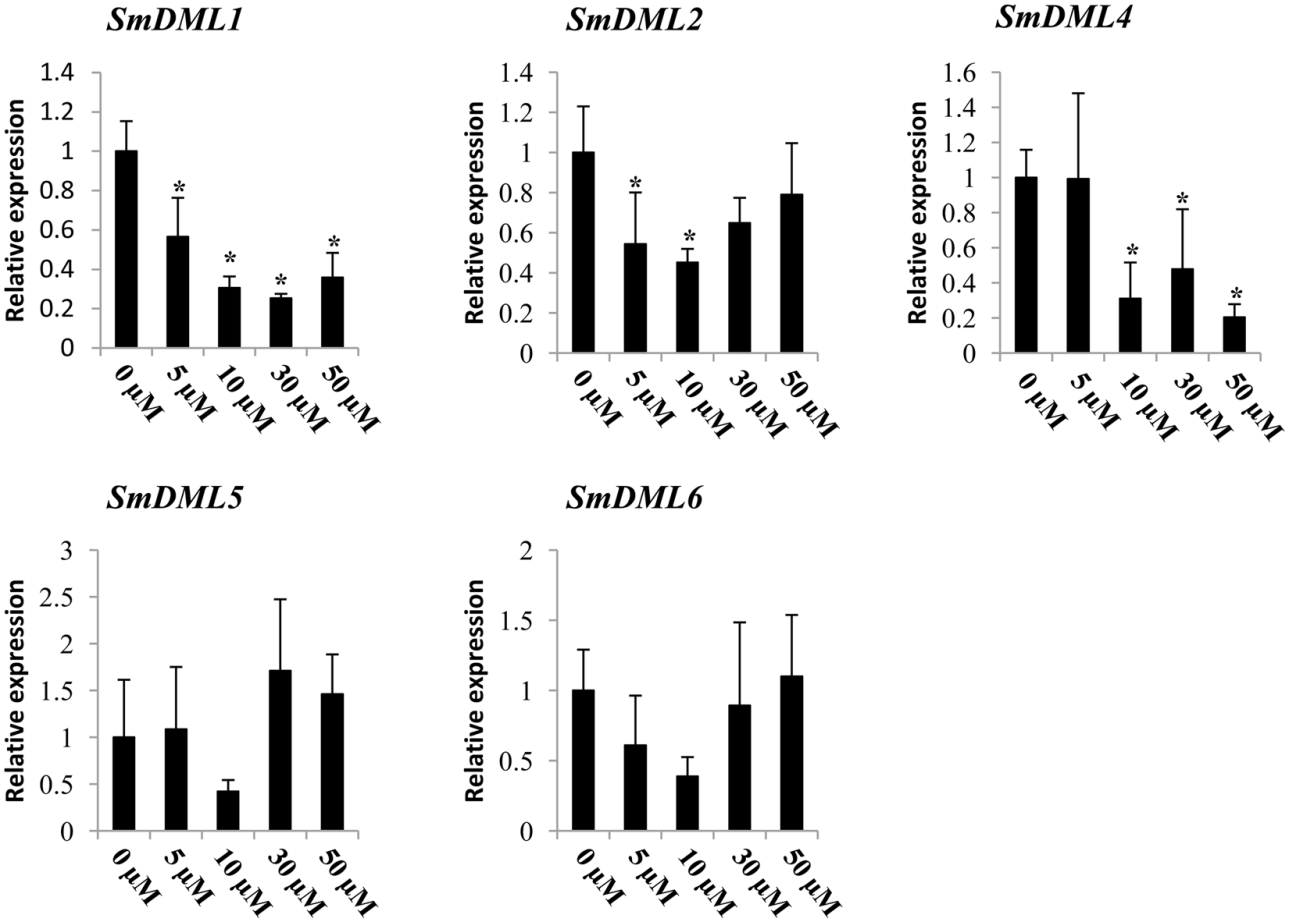
Responses of *SmDML* genes to 5Aza-C treatment. Fold changes of *SmDMLs* in leaves of *S*. *miltiorrhiza* plantlets treated with 1, 5, 10, 30 or 50 µM of 5Aza-C for 15 days are shown. The expression levels were analyzed using the quantitative RT-PCR method. Expression level in leaves without treatment (0 µM) was arbitrarily set to 1 and the levels in leaves of 5Aza-C-treated plantlets were given relative to this. One-way ANOVA was calculated using IBM SPSS 20 software. *P* < 0.05 was considered statistically significant and was represented by asterisks.

It has been shown that DNA methylation-related genes are responsive to DNA methylation inhibitor treatment and methylation-sensitive expression of *ROS1* in plants is conserved and adaptive^40^. To elucidate the expression patterns of *SmDML* genes in response to DNA methylation inhibitor treatment, we treated *S*. *miltiorrhiza* plantlets with different concentrations of the DNA methyltransferase inhibitor, 5Aza-dC. Gene expression analysis showed that *SmDML1*, *SmDML2* and *SmDML4* were significantly methylation-responsive (Fig. 6). *SmDML1* transcripts were significantly reduced regardless of the concentration of 5Aza-dC treated (Fig. 6). The expression of *SmDML2* was significantly decreased at 5 µM and 10 µM 5Aza-dC treatments; however, reduced expression of *SmDML2* was not significant at higher 5Aza-dC concentrations (Fig. 6). The expression of *SmDML4* was significantly decreased at 10 µM, 30 µM and 50 µM 5Aza-dC treatments. *SmDML5* and *SmDML6* transcript levels were not influenced by 5Aza-dC treatments, indicating they could be not involved in DNA demethylation. Interestingly, the expression of *SmDML3* was not detected in *S*. *miltiorrhiza* plantlets (Fig. 6); however, it was expressed in all mature tissues analyzed (Fig. 6), implying its important roles in *S*. *miltiorrhiza* plant maturation. Taken together, these data suggest that DNA methylation changes can influence *SmDML* expression in *S*. *miltiorrhiza*.

### miRNA-mediated posttranscriptional regulation of *SmDML1*

**Figure 7:**
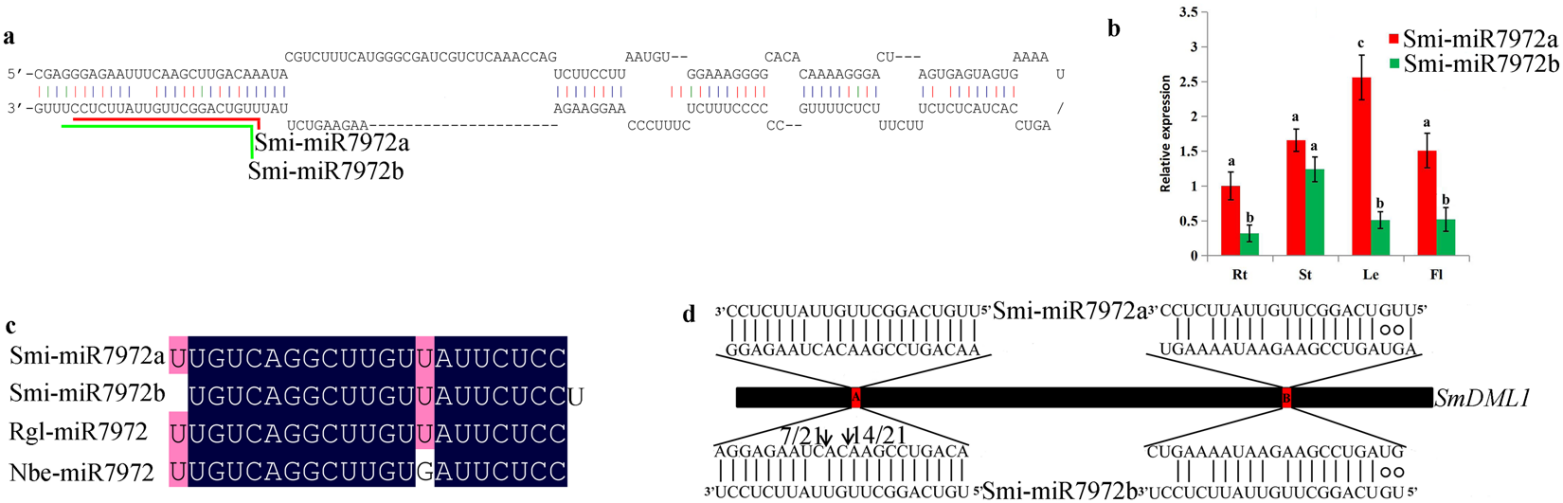
Smi-miR7972 in *S*. *miltiorrhiza*. (**a**) The hairpin structure of Smi-miR7972. Smi-miR7972a and Smi-miR7972b are indicated by red and green lines. (**b**) Expression patterns of Smi-miR7972a and Smi-miR7972b in roots (Rt), stems (St), leaves (Le) and flowers (Fl) of *S*. *miltiorrhiza*. Fold changes of Smi-miR7972a and Smi-miR7972b are shown. Expression level of Smi-miR7972a in roots was arbitrarily set to 1 and the levels of Smi-miR7972a and Smi-miR7972b were given relative to this. Error bars was indicated by the standard deviations of three biological replicates. (**c**) Sequence alignment of miR7972s from *S*. *miltiorrhiza*, *R*. *glutinosa* and *N*. *benthamiana*. (**d**) Validation of Smi-miR7972a- and Smi-miR7972b-mediated cleavage using the modified 5′ RLM RACE method. Heavy black line represents open reading frame of *SmDML1*. The complementary sites of Smi-miR7972 in *SmDML1* are represented by A and B and shown in red. The nucleotide sequences of Smi-miR7972a and Smi-miR7972b from 3′ to 5′ and the complementary sites of *SmDML1* from 5′ to 3′ are shown in the expanded regions. Vertical dashes indicate Watson-Crick pairing. Circles indicate G:U wobble pairing. Vertical arrows indicate the 5′ termini of Smi-miR7972-mediated cleavage products, as obtained by 5′ RLM-RACE, with the frequency of clones shown.

In *Arabidopsis*, *AtDML3* transcripts are cleaved by miR402^41,42^. In *Nicotiana benthamiana*, *NbROS1* is targeted by Nb_miRC1_3p^43^. In order to know whether *SmDMLs* are regulated by miRNAs, we searched high-throughput *S*. *miltiorrhiza* small RNA database for miRNAs potentially targeting *SmDMLs* using psRNATarget^44,45^. A total of 44 small RNAs with sequence reads greater than four were identified under the maximum expectation of 3.0. The retrieved small RNA sequences were aligned with *S*. *miltiorrhiza* 99-3 genome and then secondary structures of genomic DNA sequences surrounding the small RNAs were predicted and manually checked. It resulted in the identification of *Smi-MIR7972*. This miRNA gene generates two 21nt-miRNAs, termed Smi-miR7972a and Smi-miR7972b, respectively. Smi-miR7972a and Smi-miR7972b are overlapped, and Smi-miR7972a starts 1nt upstream relative to Smi-miR7972b (Fig. 7a). Quantitative qRT-PCR analysis using Smi-miR7972a- and Smi-miR7972b-specific primers showed that Smi-miR7972a was highly expressed in leaves of 2-year-old, field nursery-grown *S*. *miltiorrhiza* plants (Fig. 7b). Its expression in roots, stems and flowers is similar and relatively low compared with its expression in leaves. Smi-miR7972b showed the highest expression in stems. Its expression in roots, leaves and flowers is similar. The expression of Smi-miR7972a was higher than Smi-miR7972b in all tissues analyzed (Fig. 7b). Blast analysis of Smi-miR7972a and Smi-miR7972b against miRBase (http://www.mirbase.org/) showed that Smi-miR7972a was identical to the functionally unknown Rgl-miR7972 from *Rehmannia glutinosa*^46^. Examination of Smi-miR7972a and Nb_miRC1_3p sequences revealed that they were highly similar with only a mismatched nucleotide. It suggests that Nb_miRC1_3p is actually a member of the *MIR7972* family. Thus, Nb_miRC1_3p was renamed to Nbe-miR7972 in this report (Fig. 7c).

Computational target prediction showed that *SmDML1* contained two regions near-perfectly complementary to Smi-miR7972a and Smi-miR7972b (Fig. 7d). The regions were named region A and region B, respectively. The expectations are 1.0 and 3.0 for region A and region B, respectively, as calculated using psRNATarget^44^. To analyze the cleavage site of Smi-miR7972a and Smi-miR7972b in *SmDML1*, the modified 5′RLM-RACE experiments were carried out as previously described^47^. The results confirmed that *SmDML1* was indeed cleaved at region A *in vivo* (Fig. 7d). However, the cleavage at region B was not validated. It indicates that region B is not cleaved due to the greater expectation. It is also possible that region B is cleaved only in certain tissues or plant developmental stages. Plant miRNAs usually cleave target mRNAs at the tenth complementary nucleotide from the 5′ end of the miRNA^48,49^. Investigation of cleavage site at region A showed that there were two cleavage sites. One corresponds to the tenth complementary nucleotide from the 5′ end of Smi-miR7972b, confirming the cleavage by Smi-miR7972b. The other one corresponds to the twelfth and thirteenth complementary nucleotides from the 5′ end of Smi-miR7972b and Smi-miR7972a, respectively. It implied that this site was cleaved neither Smi-miR7972b nor Smi-miR7972a, indicating the complexity of mRNA cleavage mechanism.

### Phylogeny of *MIR7972* genes

**Figure 8:**
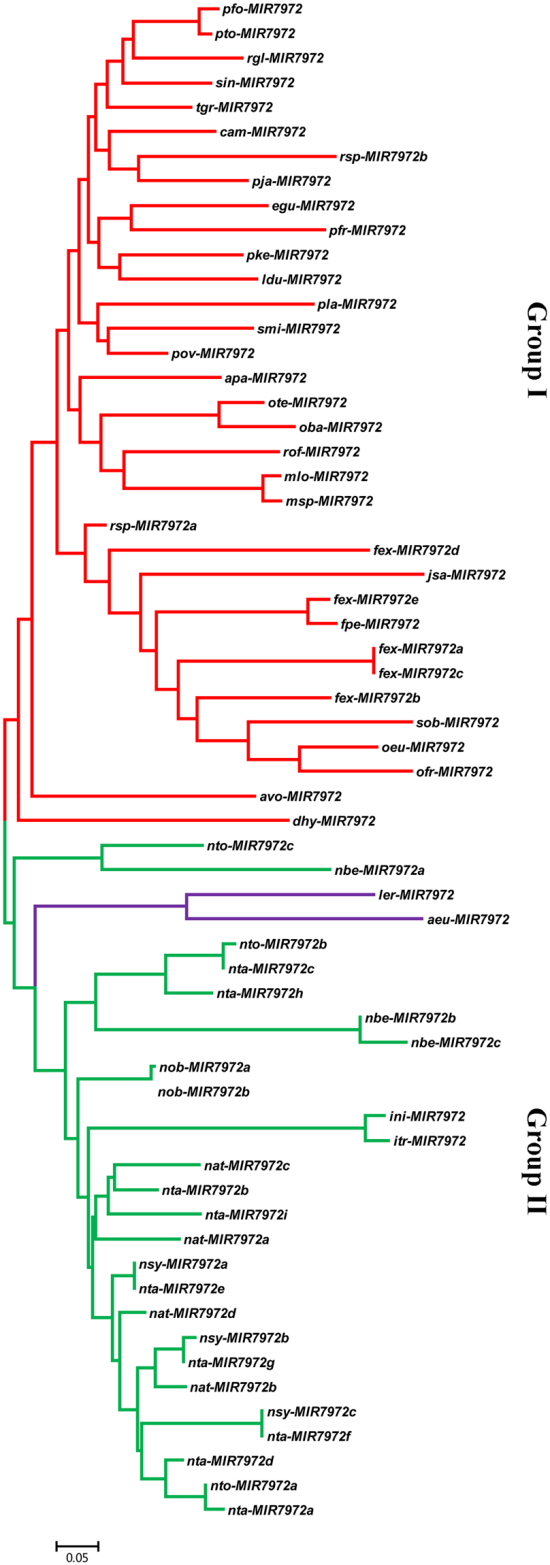
Phylogenetic relationships of MIR7972 precursors in various Lamiids species. It includes *Ruellia speciosa* (rsp), *Mentha longifolia* (mlo), *Ocimum tenuiflorum* (ote), *Fraxinus excelsior* (fex), *Dorcoceras hygrometricum* (dhy), *Sesamum indicum* (sin), *Erythranthe guttata* (egu), *Nicotiana obtusifolia* (nob), *Ipomoea nil* (ini), *Ipomoea trifida* (itr), *Nicotiana attenuata* (nat), *Nicotiana sylvestris* (nsy), *Nicotiana tomentosiformis* (nto), *Nicotiana tabacum* (nta), *Nicotiana benthamiana* (nbe), *Andrographis paniculata* (apa), *Jasminum sambac* (jsa), *Syringa oblata* (sob), *Fraxinus pennsylvanica* (fpe), *Olea europaea (oeu)*, Osmanthus fragrans *(ofr)*, *Alectra vogelii (avo)*, *Rehmannia glutinosa (rgl)*, *Phtheirospermum japonicum (pja)*, *Pedicularis keiskei (pke)*, *Conopholis americana (cam)*, *Paulownia fortunei (pfo)*, *Paulownia tomentosa (pto)*, *Plantago ovata (pov)*, *Plantago lagopus (pla)*, *Lippia dulcis (ldu)*, *Tectona grandis (tgr)*, *Ocimum basilicum (oba)*, *Perilla frutescens (pfr)*, *Rosmarinus officinalis (rof)*, *Mentha spicata (msp)*, *Lithospermum erythrorhizon (ler)*, *Arnebia euchroma (aeu)*. Species from Lamiales, Solanales and Boraginales are shown red, green and purple, respectively. *MIR7972s* could be divided into two groups, including group I and group II. The branch length is shown.

So far, miR7972 has been identified only in three plant species, including *S*. *miltiorrhiza*, *N*. *benthamiana* and *R*. *glutinosa*, all of which are placed in Lamiids. In order to know the phylogeny of *MIR7972* genes in plants, systematic and comprehensive investigation was carried out on the whole genome sequences of 40 Lamiids species (Table S4) and almost all RNA-seq data available for Lamiids species in SRA database. *MIR7972* genes exist only in Solanales, Boraginales and Lamiales. It was not found in other orders of the Lamiids clade. In total, 62 *MIR7972* genes were identified (Table S5), of which 34 were from 29 Lamiales species, 26 from 8 Solanales species, whereas the other two were from two Boraginales species. Interestingly, in Solanales, *MIR7972s* were identified only in *Nicotianeae* of Solanaceae and *Ipomoea* of Convolvulaceae. In Boraginales, *MIR7972s* were identified only in two plant species of *Lithospermum*. However, Lamiales *MIR7972s* widely exist in various families, including Oleaceae, Gesneriaceae, Plantaginaceae, Pedaliaceae, Verbenaceae, Lamiaceae, Phrymaceae, Paulowniaceae, and Orobanchaceae (Fig. 8). The majority of these families are not evolutionarily close to each other (http://www.mobot.org/MOBOT/Research/APweb/welcome.html). It indicates the complexity of *MIR7972* origin and evolution. The underlying mechanism remains to be elucidated. Most of the Lamiales species contain one *MIR7972* gene, whereas multiple copies were found in all *Nicotianeae* species, suggesting the occurrence of *MIR7972* duplication in *Nicotianeae*.

In order to reveal the phylogenetic relationship of *MIR7972s*, a neighbor-joining (NJ) phylogenetic tree was constructed for precursor sequences of the identified *MIR7972s* using MEGA7.0 (Fig. 8). *MIR7972s* could be divided into two groups. Group I contains Lamiales *MIR7972s*, whereas group II consists of *MIR7972s* from Solanales and Boraginales. It suggests closer evolutionary relationships between *MIR7972s* in Boraginales and Solanales in comparison with Lamiales. The results are consistent with previous studies showing that Lithospermeae species exhibit closer evolutionary relationships with Solanales species^50^.

## Disscussion

*S*. *miltiorrhiza* is a widely cultivated medicinal crop in East Asia and a model medicinal plant for TCM studies. It has been used as medicinal materials to treat cerebrovascular diseases and cardiovascular diseases for hundreds of years^51,52^. Many genes associated with the production of bioactive compounds, including lipid-soluble tanshinones and water-soluble phenolic acids, have been characterized^2,53,54^. However, little is known about epigenetic factors regulating gene expression in *S*. *miltiorrhiza*. Elucidation of regulatory process of DNA methylation is important for understanding gene expression regulatory mechanism associated with secondary metabolism and Dao-di herb formation in *S*. *miltiorrhiza*. In this study, six *S*. *miltiorrhiza SmDML* genes involved in DNA demethylation were identified and characterized. The number of *SmDML* genes is similar to those from castor bean, tomato, *Arabidopsis* and rice, ranging from three to six^24,55,56^.

Phylogenetic analysis of 66 DMLs from 16 plant species showed that SmDML1 was a member of the ROS1 group, SmDML2–SmDML4 belonged to the DME group, and SmDML5 and SmDML6 were included in the DML3 group (Fig. 4). The results from *Arabidopsis* and rice showed that DMLs in a group may have different functions. For instance, both rice OsROS1a and OsROS1c are members of the ROS1 group. *OsROS1a* is indispensable in both male and female gametophytes and critical to gametophytes^33^, whereas *OsROS1c* promotes expression and transposition of *Tos17*^21^. Among the three SmDMLs of the DME group, SmDML4 is the smallest with only 720 amino acids. It lacks conserved motifs 9, 10, 11, and 13 compared with SmDML2 and SmDML3 (Fig. 3). *SmDML2*–*SmDML4* showed differential expression in mature plants (Fig. 5) and *SmDML3* was not expressed in plantlets. Similarly, differential expression was observed for *SmDML5* and *SmDML6* (Fig. 5), two *SmDMLs* of the DML3 group. It indicates that *SmDMLs* from a group may also be involved in distinct biological processes.

Generally, DNA methylation suppresses gene expression, whereas DNA demethylation promotes gene expression. In this study, we found that the expression of *SmDMLs* was down-regulated after 5Aza-dC treatment. It is consistent with significant down-regulation of *DMLs* in DNA methylation mutants in *Arabidopsis*^57,58^. Low expression of *SmDMLs* may contribute to maintain the certain DNA methylation level under the presence of 5Aza-dC. Balance between DNA methylation and demethylation is important for plant growth and development^59,60^. In *Arabidopsis*, the balance is monitored by a DNA methylation monitoring sequence (MEMS) in the *ROS1* promoter region^40,60^. It is unknown whether this mechanism also exists in *S*. *miltiorrhiza*. Alternatively, *SmDMLs* were not directly regulated by DNA methylation. Down-regulation of *SmDMLs* under 5Aza-dC treatment was mediated by a complex network with various mediators.

Plant miRNAs are a class of small non-coding RNAs with about 21–22 nt in length. They play vital roles in many biological processes through RNA cleavage^61^. *Arabidopsis AtDML3* is regulated by miR402. The regulation is important for seed germination under stress conditions^42^. In *N*. *benthamiana*, miRNA-mediated repression of *ROS1* may strength transcriptional gene silencing^43^. Although *S*. *miltiorrhiza* miRNAs have been reported^3,45,62–65^, their functions are largely unknown. Analysis of high-throughput sRNA data allowed us to identify Smi-miR7972a and Smi-miR7972b targeting *SmDML1* for cleavage. It indicates that miRNAs play significant roles in the regulation network of DNA methylation in *S*. *miltiorrhiza*. Smi-miR7972b accumulated at lower levels than Smi-miR7972a in the tissues analyzed (Fig. 7b). The variance between the levels of Smi-miR7972a and Smi-miR7972b could be caused by differential sequence preference of DICER-LIKE 1 (DCL1) responsible for miRNA biogenesis^66,67^. Experimental evidence showed that the cleavage of *SmDML1* was mediated by Smi-miR7972b rather than Smi-miR7972a in the tissues analyzed (Fig. 7d). The possibility that Smi-miR7972a cuts *SmDML1* in other tissues cannot be ruled out. Alternatively, Smi-miR7972b is easier to be recruited by AGO proteins for the formation of RNA induced silencing complex (RISC)^68^.

*MIR7972* genes were only identified in some species of the three Lamiids orders, including Solanales, Lamiales and Boraginales, and the number of *MIR7972* genes varied among species. The actual origin and evolution mechanism of *MIR7972* is unknown. One of the possibilities is that the *MIR7972* genes in different plant species were originated from a common ancestor of Solanales, Lamiales and Boraginales. Loss and duplication of *MIR7972* occurred in some plant species during evolution. This possibility is consistent with frequent birth and death of some *MIRNA* genes^69^. Independent origin and evolution of *MIR7972s* in different lineage is the other possibility. Evidence to support this possibility is that some miRNAs evolved are rarely lost and highly conserved across taxa^70^. The gain and loss of *MIR7972* could be important for plants of some lineages to survive in the stressful environments. Further investigating the biological function of *MIR7972* will help to elucidate the evolution mechanism of *MIR7972*.

## Materials and Methods

### Plant materials

*Salvia miltiorrhiza* Bunge (line 99-3) plants were cultivated in a field nursery at the Institute of Medicinal Plant Development, Beijing, China. Roots, stem, leaves and flowers of two-year-old plants were collected and stored immediately in liquid nitrogen until use. For 5-aza-2′-deoxycytidine (5Aza-dC) treatment, plantlets were grown on Murashige and Skoog (MS) agar medium^71^ supplemented with 0, 5, 10, 30 or 50 µM 5Aza-dC (Sigma) for 15 days at 25 °C under a photoperiod of 16 h light and 8 h dark. Newly generated leaves were collected and immediately stored in liquid nitrogen until use. Three independent biological replicates were carried out for each treatment.

### *SmDML* gene identification

The deduced amino acid sequences of four *Arabidopsis* DML proteins were downloaded from the TAIR database (http://www.arabidopsis.org). To predict *SmDML* genes, AtDMLs were used as queries to search the two databases of *S*. *miltiorrhiza* whole genome sequence^6,7^. The searches were carried out using the tBLASTN program^72^. An e-value cut off of 1e^−10^ was applied. Gene models were predicted on the GENSCAN web server (http://genes.mit.edu/GENSCAN.html) for retrieved genomic DNA sequences^34^. The predicted gene models were then manually examined and corrected by comparison with *DML* genes identified from other plants using the BLASTx algorithm (www.ncbi.nlm.nih.gov/blast/) and by alignment with RNA-seq data of *S*. *miltiorrhiza* transcriptome (http://www.ncbi.nlm.nih.gov/sra). The INTERPRO database (http://www.ebi.ac.uk/interpro/) was finally used to confirm each predicted protein sequence to be a DML.

### Gene structure and protein sequence analysis

Gene structures of *SmDMLs*, *AtDMLs* and *OsDMLs* were determined on the Gene Structure Display Server (GSDS 2.0; http://gsds.cbi.pku.edu.cn/index.php). Coding sequences and corresponding genomic sequences were used as inputs. The deduced protein sequences of SmDMLs, AtDMLs and OsDMLs were analyzed for amino acid number, molecular weight (Mw), theoretical isoelectric point (p*I*) using the EXPASY PROTOPARAM tool (http://www.expasy.org/tools/protparam.html). Multiple sequence alignment was performed for SmDML, AtDML and OsDML amino acid sequences using ClustalW. Conserved motifs in SmDML, AtDML and OsDML proteins were detected using the MEME suite (http://meme.sdsc.edu/meme/meme.html).

### Phylogenetic analysis

Unrooted neighbor-joining (NJ) trees were constructed using MEGA (version 7.0) with 1000 bootstrap replicates^73^. Protein sequences of DMLs from 16 plant species were downloaded from Phytozome (http://phytozome.jgi.doe.gov/pz/portal.html) (Table S1). Ka and Ks values were calculated for two gene pairs, *SmDML2*/*SmDML3* and *SmDML5*/*SmDML6*, using DNASP5 software^74^.

### RNA extraction and qRT-PCR analysis

Total RNA was isolated from *S*. *miltiorrhiza* tissues using the plant total RNA extraction kit (Aidlab, China). The isolation was carried out following the manufacturer’s instructions. RNA integrity was analyzed on an agarose gel. RNA quantity was determined using a NanoDrop 2000C spectrophotometer (Thermo Scientific, USA). Reverse transcription was conducted using PrimeScript™ RT reagent kit (TaKaRa, Japan). Gene specific primers were designed using Primer Premier 6 (PREMIER Biosoft Int, USA) based on *SmDML* coding sequences. *SmUBQ10* was used as an internal control as described previously^2^. The expression of Smi-miR7972a and Smi-miR7972b was analyzed using Mir-X miRNA qRT-PCR SYBR Kit (TaKaRa, Japan). The primers were listed in Table S2. qRT-PCR was performed in triplicate for each tissue sample using the SYBR premix Ex Taq™ kit (TaKaRa, China) on a CFX96 Touch™ real-time PCR system (Bio-Rad, USA). Three independent biological replicates were performed. Gene relative expression levels were calculated for Ct values using the 2^−ΔΔCq^ method^75^. Differential expression among tissues and treatments was determined by one-way ANOVA using IBM SPSS 20 software (IBM Corporation, USA).

### RAN-Seq data and bioinformatic analysis

Transcriptome sequencing data generated for periderm, phloem and xylem of *S*. *miltiorrhiza* roots was downloaded from SRA database of NCBI (SRX751296)^54^. Differential expression of *SmDML* genes was analyzed using TopHat2.0.12 and Cufflinks2.2.1^39^.

### Identification of *S*. *miltiorrhiza* miRNAs potentially targeting *SmDMLs*

*S*. *miltiorrhiza* small RNAs potentially targeting *SmDMLs* for cleavage were predicted using psRNATarget^44^. Small RNAs from roots, stems, leaves and flowers of *S*. *miltiorrhiza* were downloaded from SRA database (SRX686651, SRX686652, SRX686653 and SRX686654)^45^. The maximum expectations of 3.0 and the target accessibility-allowed maximum energy to unpair the target site of 25 were applied. The predicted small RNAs were mapped to *S*. *miltiorrhiza* 99-3 genome using Bowtie^76^. No mismatch was allowed. Secondary structures of genomic sequences surrounding small RNA-aligned regions were predicted on the mfold web server^77^. The structures were manually checked and miRNAs were annotated under the criteria described^70^.

### 5′ RLM-RACE validation of miR7972-directed cleavage

Roots, stems, leaves and flowers of two-year-old *S*. *miltiorrhiza* were used for validation of miR7972-directed cleavage. The modified RNA ligase-mediated rapid amplification of 5′ cDNAs (5′ RLM-RACE) was carried out using the FirstChoice^®^ RLM-RACE Kit (Invitrogen, Carlsbad, CA). The nesting and nested primers were 5′-GGGGCAACCTGGTGAGATTCCATCT-3′ and 5′-ACCGGTTAACACCATTTTTCCGA-3′, respectively. Nesting PCR was carried out under the touchdown conditions: 94 °C for 3 min, 5 cycles of 94 °C for 30 s and 72 °C for 90 s, 5 cycles of 94 °C for 30 s, 70 °C for 30 s and 72 °C for 50 s, 25 cycles of 94 °C for 30 s, 60 °C for 30 s and 72 °C for 1 min, followed by a final extension at 72 °C for 10 min. Nested PCR amplification was performed under following conditions: 94 °C for 3 min, 35 cycles of 94 °C for 30 s, 60 °C for 30 s and 72 °C for 30 s, followed by a final extension at 72 °C for 10 min.

### Identification of *MIR7972* genes in Lamiids

*MIR7972* precursors from *S*. *miltiorrhiza*, *Rehmannia glutinosa* and *Nicotiana benthamian* were used to blast genomes of 40 Lamiids plant species listed in Table S3 using BLASTn^72^. Transcriptome-wide identification of *MIR7972* was performed through BLAST analysis of *Smi-MIR7972* or *Nbe-MIR7972* against RNA-seq reads (https://www.ncbi.nlm.nih.gov/sra) from Lamiids plants using BLASTn^72^.

## Electronic supplementary material

Supplementary Information
