# Supplementary material for: Systematic analysis of DEMETER-like DNA glycosylase genes shows lineage-specific Smi-miR7972 involved in SmDML1 regulation in Salvia miltiorrhiza
Source: Sci Rep. 2018 May 8;8:7143. doi: 10.1038/s41598-018-25315-w (PMC5940787; doi:10.1038/s41598-018-25315-w)
Supplement: Supplementary file 1 — Supplementary Information [file 41598_2018_25315_MOESM1_ESM.pdf]

Systematic analysis of *DEMETER*-like DNA glycosylase genes shows lineage-specific Smi-miR7972 involved in *SmDML1* regulation in *Salvia miltiorrhiza*

Jiang Li, Caili Li, Shanfa Lu*

Institute of Medicinal Plant Development, Chinese Academy of Medical Sciences & Peking Union Medical College, No.151 Malianwa North Road, Haidian District, Beijing 100193, China

*Corresponding author
Shanfa Lu
Tel: +86-10-57833366
Fax: +86-10-57833366
Email: sflu@implad.ac.cn

Supplementary Table S1. *DML* genes identified in other plant species.

Genename	Gene model
AtDME	Ath_AT5G04560
AlDME	Aly_AL6G14210.t1
PtDME1	Potri.010G234400.1
PtDME2	Potri.008G025900.1
MeDME1	Manes.07G091800.1
MeDME2	Manes.10G055200.1
RcDME	29428.m000327
MgDME1	Migut.F00888.1
AtROS1	AT2G36490.1
AlROS1a	AL4G32680.t1
AlROS1b	AL4G32670.t1
AtDML2	AT3G10010.1
AlROS1c	AL3G21300.t1
PtROS1	Potri.006G116000.1
RcROS1	29092.m000452
MeROS1a	Manes.09G060800.1
MeROS1b	Manes.08G020500.1
MgROS1	Migut.J00987.1
OsROS1d	Os05g37410.1
OsROS1c	Os05g37350.1
SbROS1a	Sobic.009G155900.1
BdROS1a	Bradi2g23797.4
BdROS1b	Bradi4g16620.5
ZmROS1a	GRMZM2G131756_T01
BdROS1c	Bradi4g08870.2
OsROS1a	Os01g11900.1
BdROS1d	Bradi3g43692.1
OsROS1b	Os02g29230.1
SbROS1b	Sobic.004G149800.1
SbROS1c	Sobic.008G085300.1
ZmROS1b	GRMZM2G422464_T02
MgDML3	Migut.D02337.1
MgDME2	Migut.L00609.1
AtDML3	AT4G34060.1
AlDML3	AL7G17320.t1
PtDML3	Potri.001G150000.1
MeDML3	Manes.04G008900.1
RcDML3	29991.m000647
OsDML3b	Os04g28860.1
BdDML3	Bradi3g43720.1
OsDML3a	Os02g29380.1
ZmDML3	GRMZM5G828460_T01

SbDML3	Sobic.006G224100.1
CsDML3	Cucsa.385370.1
CsROS1a	Cucsa.083110.1
CsROS1b	Cucsa.378580.1
CsDME	Cucsa.308950.1
SlROS1a	Solyc10g083630.1.1
SlDML3	Solyc03g123440.2.1
SlROS1b	Solyc09g009080.2.1
SlDME	Solyc11g007580.1.1
GmDME	Glyma.20G188300.1
GmROS1b	Glyma.10G065900.1
GmROS1c	Glyma.13G151000.1
GmROS1a	Glyma.03G190800.1
ClDME	Ciclev10030474m
ClROS1	Ciclev10010892m
ClDML3	Ciclev10027676m
VvROS1	GSVIVT01033777001
VvDME	GSVIVT01034713001

Supplementary Table S2. The primers for qRT-PCR analysis

Gene	Forward primer	Reverse primer
SmDML1	TTCCCTGCCAGCAAGCTAGC	AGCCGCTCACTGTTCAACCA
SmDML2	TCGGCAACCAAGGAGAAGCAA	GTGCCTTTTGAGGTGGTGTGC
SmDML3	GGCTTGGCTTGGCTTTTCTGG	TCACCCCATCAACTGCTTGCA
SmDML4	ATTGCTGTGAGTGCCCGATGA	GTCCATTTCAGCGAGCCCTCT
SmDML5	GCAATTCTCGCCGTGGAAAGG	ATGACAAAGCCATGAACGCCG
SmDML6	CGCCATAAGAAGCCGCACAAG	CACTTTCTTGGCGGGCTTTGG
Smi-miR7972a	TTGTCAGGCTTGTTATTCTCCA	
Smi-miR7972b	TGTCAGGCTTGTTATTCTCCT	

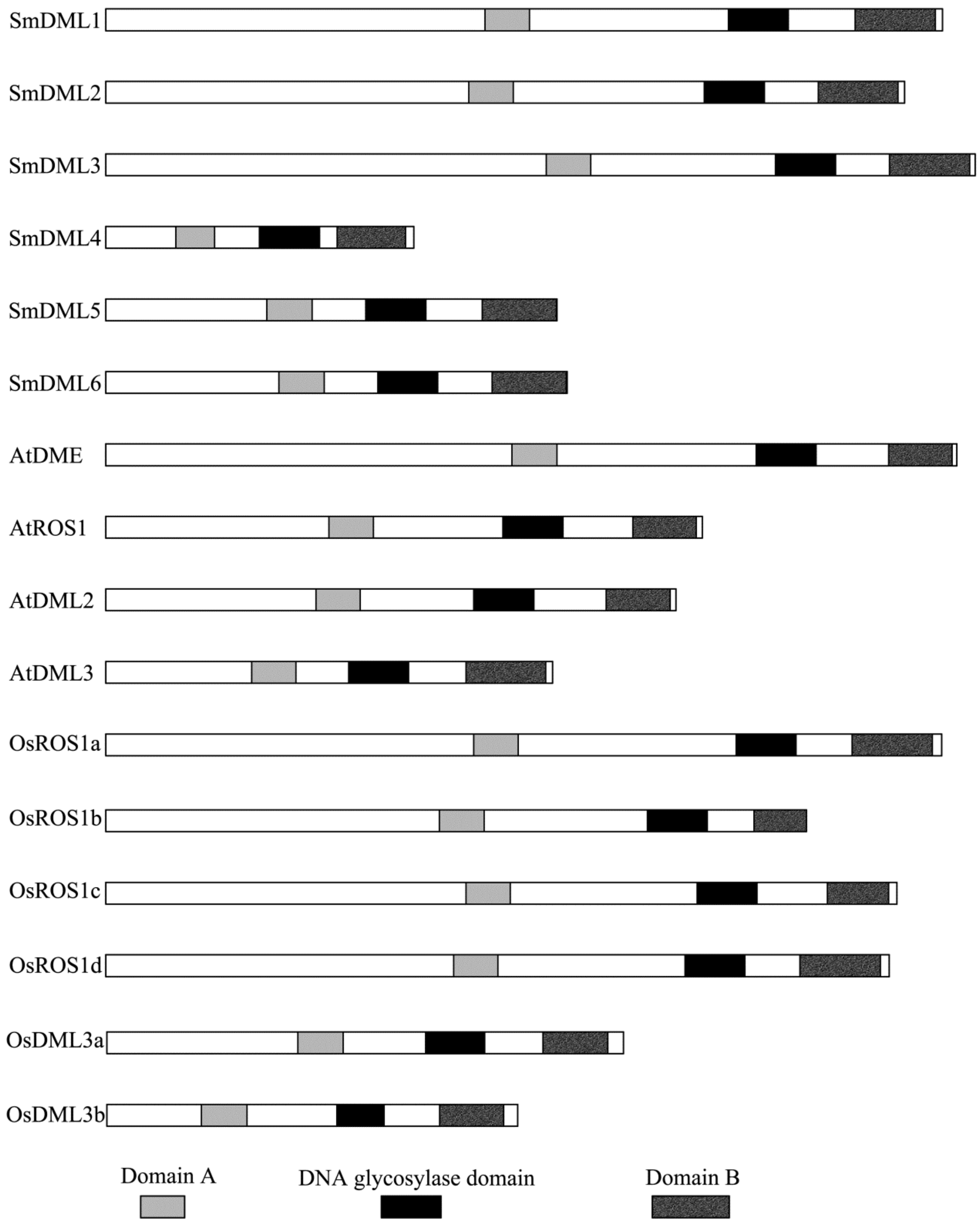

Supplementary Figure 1. Schematic view of DML proteins from *S.miltiorrhiza*, *Arabidopsis* and rice.

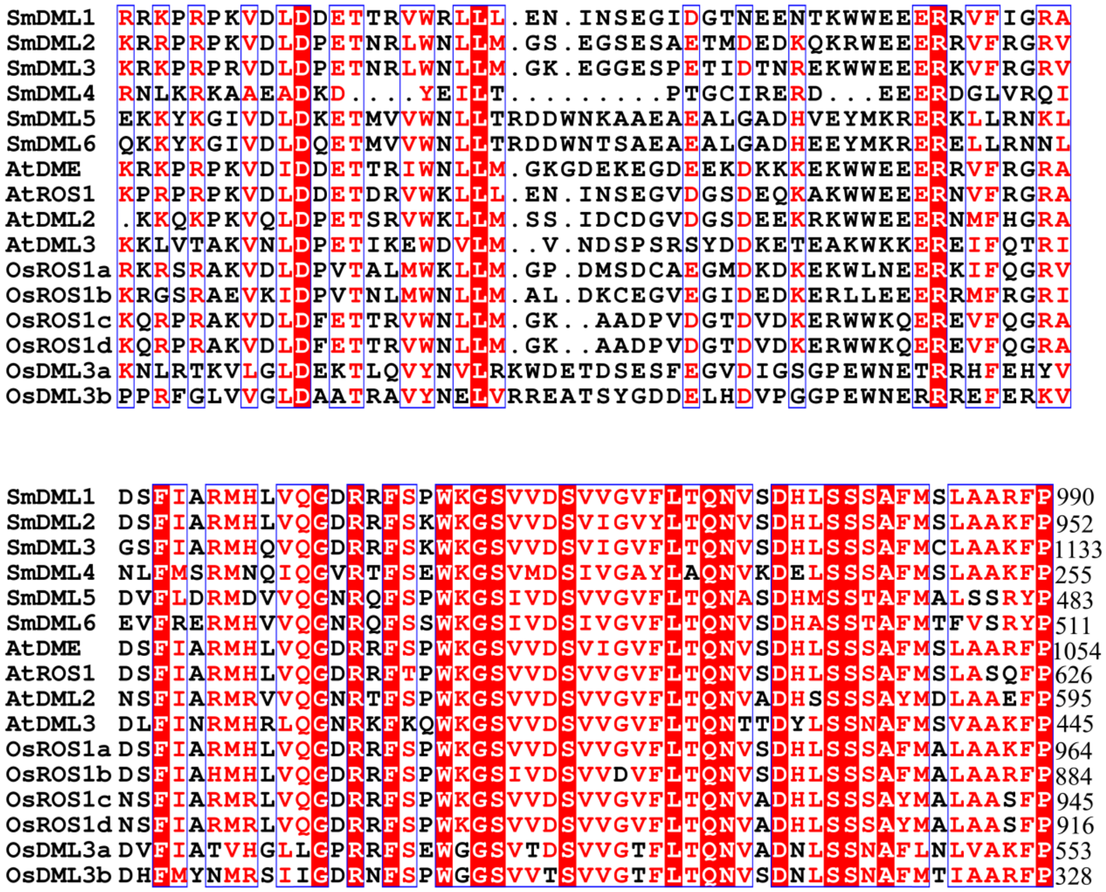

Supplementary Figure 2. Amino acid sequence alignment of the conserved domain A of DML proteins in *S. miltiorrhiza*, *Arabidopsis* and rice shows amino acid positions (numbers) and identical(red background) or similar (red)amino acids.

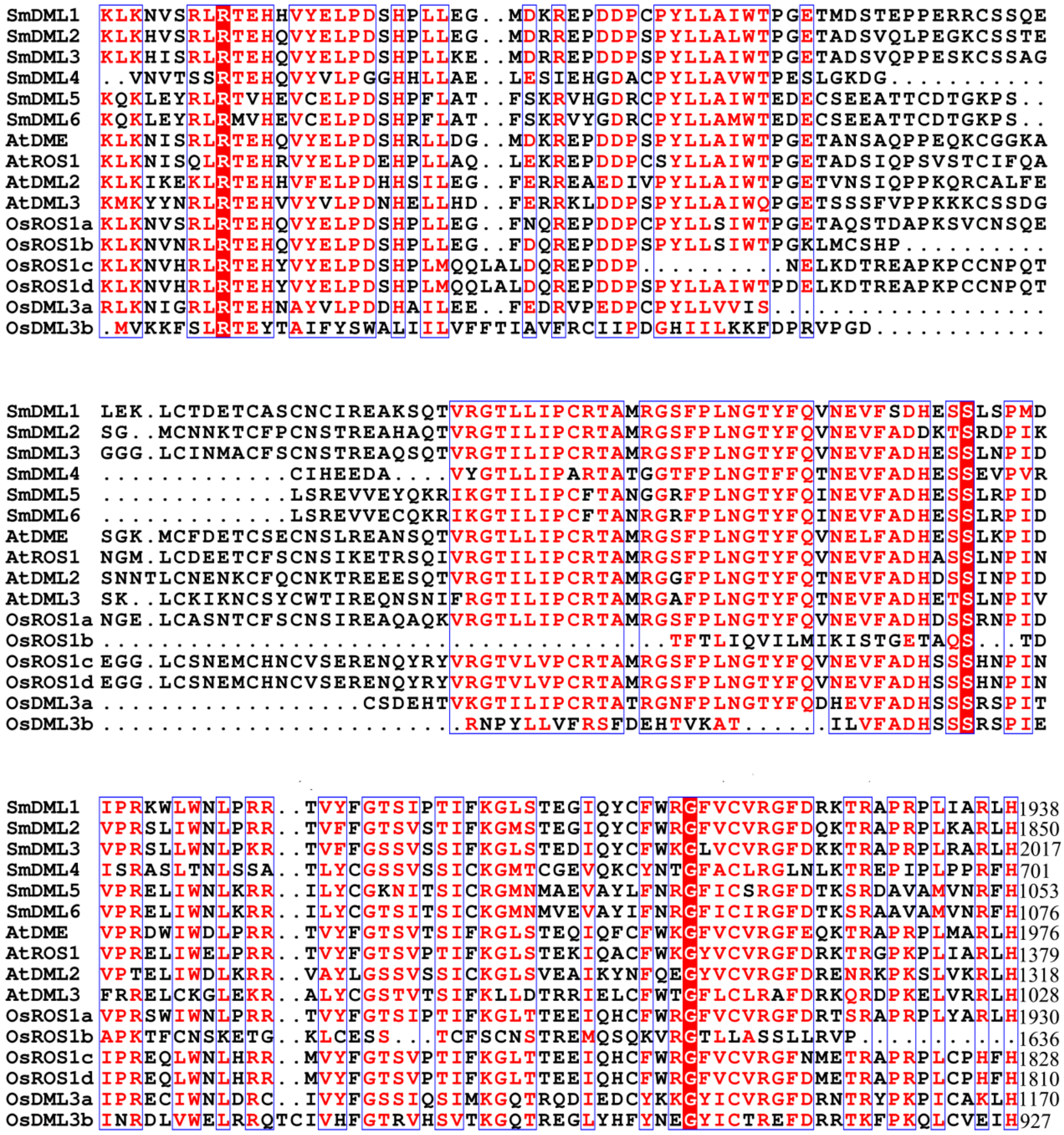

Supplementary Figure 3. Amino acid sequence alignment of the conserved domain B of DML proteins in *S. miltiorrhiza*, *Arabidopsis* and rice shows amino acid positions (numbers) and identical (red background) or similar (red) amino acids.

Supplementary Table S3. The expression (FPKM) patterns of *SmDMLs* in the periderm, phloem and xylem of *S. miltiorrhiza* roots.

Gene	Periderm	Phloem	xylem
SmDML1	17.77	23.39	28.44
SmDML2	5.35	10.08	7.68
SmDML3	12.27	18.02	17.88
SmDML4	0.03	0.29	0.16
SmDML5	0.02	0.06	0.00
SmDML6	0.07	0.13	0.06

Supplementary Table S4. The genome data from 40 Lamiids species.

Lamiids species	Genome
Capsicum annuum	https://www.ncbi.nlm.nih.gov/genome/10896
Capsicum baccatum	https://www.ncbi.nlm.nih.gov/genome/56712
Solanum americanum	https://www.ncbi.nlm.nih.gov/genome/55990
Nothapodytes nimmoniana	https://www.ncbi.nlm.nih.gov/genome/54072
Nicotiana obtusifolia	https://www.ncbi.nlm.nih.gov/genome/53488
Ruellia speciosa	https://www.ncbi.nlm.nih.gov/genome/50955
Ipomoea nil	https://www.ncbi.nlm.nih.gov/genome/46552
Capsicum chinense	https://www.ncbi.nlm.nih.gov/genome/45488
Mentha longifolia	https://www.ncbi.nlm.nih.gov/genome/44852
Ocimum tenuiflorum	https://www.ncbi.nlm.nih.gov/genome/40058
Solanum commersonii	https://www.ncbi.nlm.nih.gov/genome/39785
Ipomoea trifida	https://www.ncbi.nlm.nih.gov/genome/37016
Penstemon grinnellii	https://www.ncbi.nlm.nih.gov/genome/32664
Penstemon centranthifolius	https://www.ncbi.nlm.nih.gov/genome/32663
Nicotiana otophora	https://www.ncbi.nlm.nih.gov/genome/32281
Solanum arcanum	https://www.ncbi.nlm.nih.gov/genome/31602
Fraxinus excelsior	https://www.ncbi.nlm.nih.gov/genome/31117
Genlisea aurea	https://www.ncbi.nlm.nih.gov/genome/24580
Solanum habrochaites	https://www.ncbi.nlm.nih.gov/genome/24151
Solanum pennellii	https://www.ncbi.nlm.nih.gov/genome/24150
Rhazya stricta	https://www.ncbi.nlm.nih.gov/genome/22340
Utricularia gibba	https://www.ncbi.nlm.nih.gov/genome/16713
Penstemon fruticosus	https://www.ncbi.nlm.nih.gov/genome/13612
Penstemon dissectus	https://www.ncbi.nlm.nih.gov/genome/13465
Penstemon davidsonii	https://www.ncbi.nlm.nih.gov/genome/13464
Penstemon cyananthus	https://www.ncbi.nlm.nih.gov/genome/13453
Nicotiana attenuata	https://www.ncbi.nlm.nih.gov/genome/13243
Nicotiana sylvestris	https://www.ncbi.nlm.nih.gov/genome/13135
Catharanthus roseus	https://www.ncbi.nlm.nih.gov/genome/12882
Nicotiana tomentosiformis	https://www.ncbi.nlm.nih.gov/genome/12239
Dorcoceras hygrometricum	https://www.ncbi.nlm.nih.gov/genome/12223
Sesamum indicum	https://www.ncbi.nlm.nih.gov/genome/11560
Asclepias syriaca	https://www.ncbi.nlm.nih.gov/genome/11317
Solanum pimpinellifolium	https://www.ncbi.nlm.nih.gov/genome/11283
Solanum melongena	https://www.ncbi.nlm.nih.gov/genome/10973
Nicotiana benthamiana	https://www.ncbi.nlm.nih.gov/genome/10940
Erythranthe guttata	https://www.ncbi.nlm.nih.gov/genome/497
Nicotiana tabacum	https://www.ncbi.nlm.nih.gov/genome/425
Solanum tuberosum	https://www.ncbi.nlm.nih.gov/genome/400
Solanum lycopersicum	https://www.ncbi.nlm.nih.gov/genome/7

Supplementary Table S5. *MIR7972* precursor sequences identified from Solanales, Boraginales and Lamiales.

>*smi-MIR7972*
CGATCAGATCTGGTCCCTTCTCCTATTTAGATGCGGAGTAGGTTCATATCTTC
GCCTAATACTTTTTTCAGACTGAATCGAGGGAGAATTTCAAGCTTGACAAAT
ACGTCTTTCATGGCGATCGTCTCAAACCAGTCTTCCTTAATGTGGAAAGGG
GCACACAAAAGGGACTAGTGAGTAGTGAAAATAGTCCACTACTCTCTTTCT
TTCTCTTTTGCCCCCCTTTCTCTTTCCCAAGGAAGAAAGAAGTCTTATTTGT
CAGGCTTGTTATTCTCCTTTGATGGCGTGAAATGGTAAGAGGCGGTGATTG
GATGCCTCCGCGTCTGAGTTTGAGAATGGAACAGATCTCTGATCG
>*rsp-MIR7972a*
ATCAAAGGAGAATTTCAAGCTTGATTAATGCTAATTCGAAGAAGATTTGCT
GAATCTTGTTCGTCAGGCTTGGCATTCTCCTTTGAT
>*rsp-MIR7972b*
TCGAAGGAGAATTTCAAGCTGACAAATACGTCTTCCATGGCGTCCTCTGAA
ACCCACTTTGCGATTATATGCAAATACTACATAAAACATTACTAAAACGGTC
TTCAATACCCAGTCAGGCTTCAGGACAAGGATTGGCCGGCCCGACCCGAC
CCAGCCCGATCTCATTTGTCAGGCTTGTTATTCTCCTTCGG
>*mlo-MIR7972*
GTCTATATCTTCGTCTGATACTTTTTCAGGTCAAATCGAAGAAGAATTTCAA
GCTTGACAAATACGTCTAACGGCGGCCCTGCGGAAACCAGTCTTCCTTAAT
ATGGATAGGGGCAAACAAAAGGGGATTAGTCAAAATAGTCAACACTTCTCT
CTCTCTCTCGTTTGCCCCTTTCTCTCTTATAAGGAAGCAGTCTTATTTGTCAG
GCTTGTTATTCTTCTTTGATGGCGTGAAAATGGTAGGAGGCGGCGATTGGAT
>*ote-MIR7972*
TTTCACGTTGAATCGAATGAGAATTTCAAGCTTGACAAATACGTGGGACTT
GCCGTCGTTCCGAAAGCAGCCTTCCTTAACGAGAGAATATCGGCACAAATA
CTCCCTTTCTACCATTTCTCTCTCTTGAGGAAGCAGTCTTATTTGTCAGGCT
TGTTATTCTCCTTTGATGGCGTGAAA
>*fex-MIR7972a*
GAGGGAGAATTACAAGCTTGATAAATGAGTTAGCCGTCTGAAGCATTCGTT
TCGAAAATAGCGCATATCCAGTGATATACGCGTACTAAAACACGTAGTTAGT
ACTATCATATAAAGGCAAACTCTTTTGTCAGGCTTGTCATTCTCCTTT
>*fex-MIR7972b*
TTCGATGTCAATTGAGGGAGAATTACAAGTTTGACAAACGAGTTTTCCTCC
GCCCATTCTATAAACTACATGTCGCCCACCCAGTAGTGTTACACACCTAACT
AGCACTATTATAAAAGGTGAACTCTTTTGTCAGGCTTGTCATTCTCCTTTGT
TGGCATCGAA
>*fex-MIR7972c*
GAGGGAGAATTACAAGCTTGATAAATGAGTTAGCCGTCTGAAGCATTCGTT
TCGAAAATAGCGCATATCCAGTGATATACGCGTACTAAAACACGTAGTTAGT
ACTATCATATAAAGGCAAACTCTTTTGTCAGGCTTGTCATTCTCCTTT
>*fex-MIR7972d*

TTTTTATGTTAAATCGAAGTAGAATTACAAGCTTGATAAATGAGTCGGACAT
GGAGTCAGAAACGAGTTTGTGACTACAGGCGCAGATACACTCACTTTAAC
ACACTTTACTAGCACTATAAAAGCCGACTTTTTGTCAGGCTTGTAATTCTCC
TTCGGTGGCATAAAA
>*fex-MIR7972e*
TTCGATGATATCGATGGAGAATTACAAGTTTGACAAATGAGTTTTTCACAGC
GTCCTGAATTAGTTCTAAACTACAGGGCGCACAGTAGTGCTAGATACCTAA
CTAGCTCTATTTATGAAAGGGAAGCTCTTTTGTCAGGCTTGTCATTCTCCTT
TGCTGGCATCGAA
>*dhy-MIR7972*
TTCATATTTTTTTACGTCGATTCGAAGGAGAATCGCAAGCCTAACAAATCT
GAATCGAGAAATGCCTCGAGATATTCTTCTTTTGATGTGGAGAGGGTACGC
AAAAGTAGAATTGGATCATATTTGTCAGGCTTGTTATTCTCCCTCGACGCCG
TGAGAAAACGTAACATGTGGG
>*sin-MIR7972*
TTTCAGGCTGAATCGAGGGAGAATTTCAAGCTTGACAAATACGTCTTTCAT
GGCGTCCGTCCGAAACCAGTCTTATGTGGAAAGGGCACACTCAATGAATG
GATAGACTAGTAAAATAGTCAAAAACAAGTGAGTAGTGCTATTTCCCCTATT
ATTAAGGAGGCTGGCTGAGTCTTATTTGTCAGGCTTGTCATTCTCCTTTGAT
GGCGTGAAA
>*egu-MIR7972*
TTTCGCACCGAATCGAAGGAGAATTTCAAGCTTGACAGATACGTCTTTCAT
GGCGTCCGTCCGAAAACCAACCTTCCTTCCTTGATAGTGGAAAGGGTACAC
GCAATAAAATAGAAAGACTAGTAAAATAGCAAAGCTTACTTCTTTCCACTTT
ATCTTCAAGGAAGGCCGAGTCTTATTTGTCAGGCTTGTCATTCTCCTTCGAT
GGCGCGAAA
>*nob-MIR7972a*
GAAGGAGAATTACAAAGCTGACAAATACGTTTTGACGCTGATGTTTCTTGT
AATAAAGTTACTCTTTTTTAACAAGAGACATGTTCTTTCCGTTTTTGGAAAG
AGACATATTTGTCAGGCTTGTGATTCTCCTTC
>*nob-MIR7972b*
GAAGGAGAATTACAAAGCTGACAAATACGTTTTGACGCCGATGTTTCTTGT
AATAAAGTTACTCTTTTTTAACAAGAGACATGTTCTTTCCGTTTTTGGAAAG
AGACATATTTGTCAGGCTTGTGATTCTCCTTC
>*ini-MIR7972*
TTTCGTCTCATCGAAAAAGAATTACAATCCTGACAAATTCGTCGCCGGCGT
CTTTTTCTCAAGAATTTGCCGGACCATATTTTGCAGAGACATACCTTTAATCT
TTGAAGTTCATCAATTCCCAAAGTTTTTTCTCTATTCTTAATTTTCCTCCCTA
AATTTTTGGGAAAGGACCCGTTAGAAGTGACGGATTTGTCAGGCTTGTGGT
TCTTTTCCGTTGAGCTGAAA
>*itr-MIR7972*
TTTCGTCTCATCGAAAAAGAATTACAATCCTGACAAATTCGTCGCCGGCGT
CTTTTCTCAAGACTTTGCCGGACCATATTTTGCAGAGACACACCTCTAATCT
TTGATGTTCATCAATTCCCAATTTTTTAAACCTATTCTTATTTTTCCTCCCTAA

ATTTTTGGGAAAGGACCCGTTAGAAGTGACGGATTTGTCAGGCTTGTGGTT
CTTTTCCGTTGAGCTGAAA
>*nat-MIR7972a*
AAGGAGAATTACAAATCTGACAAATAAATTTTCGTCCGAGTTCTTTTAATAA
AGTTTTCTTTCTTGCAAAGAGACATGTTCTTTCCGTTTTTGGAAAGTGACAT
ATTTGTCAGGCTTGTAATTCTCCTT
>*nat-MIR7972b*
AGGAGAATTACAAATCTGACAAATAGGTTTTATCGCGCCCGAGTTTCCTTTA
ATAAAGTTCTTCTTTCTTAAAAAGAGACATGTTCTTTCCGTTTTTGGAAAGT
GACATATTTGTCAGGCTTGTGATTCTCCT
>*nat-MIR7972c*
GAGGGAGAATTACAAAACTAACAAATACATTTACATGCCGTCCCGGTTTCT
TTTAATAAAGTTCCTCTTTCTTAAAGAGACACGTTCTTTCAAATTTTGGAAA
GTGACATATTTGTCAGGCTTGTGATTCTCCTTC
>*nat-MIR7972d*
GAAGGAGAATTACAAATCTGACAAATATGTTTCGGCGTCTGGGGTTTCTTTA
ATAAAGTTCTTCTTTCTTAAAAAAGACATGTTCTTTCCATTTCTCGAAAGTG
ACATATTTGTCAGGCTTGTGATTCTCCTTC
>*nsy-MIR7972a*
GAAGGAGAATTACAAATCTGACAAATAGGTTTCGGCGTCCCGGATTTCCTT
AATAAAGTTCTTTTTTCTTAAAAAAGACATGTTCTTTCCGTTTTTGGAAAGT
GACATATTTGTCAGGCTTGTGATTCTCCTTC
>*nsy-MIR7972b*
ATTTTCTTGTCGAAGGAGAATTACAAATCTAACAAATACACCAATTTCCTAT
AATAAAGTTCTTCTTTCTTAGAAAGAGACATGTTCTTTCCGTTTTTGGAAAG
TGACATATTTGTCAGGCTTGTGATTCTCCTTTGATGGCATAGAAAAT
>*nsy-MIR7972c*
TGATATAATTTTCTTGTTATGTAGAAGGAGAATTATAAGTCTTGCAATTAGGT
TTCGGTATCGCGGGTTTCCTTTAATAAATTTCTTCTTTCTTAAAAAAGACATG
TTCTTTCCTTCTTGGTTCCGTTTTTGGAAAGTGACATATTTGTCAGGCTTGT
GATTCTCCTTCCATGACACAGAAAATGGTACCA
>*nto-MIR7972a*
GAAGTAGAATTACAAATCTGCCAAATAAGTTTCGCGTTTCTTTTAATAAAGT
TACTCTTTCTTAAAAAAGAGACATGTTCTTTCCGTTTTTGGAAAGTGACATA
TTTGTCAGGCTTGTGATTCTCCTTC
>*nto-MIR7972b*
TTATCGAATGATACAATTTTCTTGTTATATATAAGGAGAATTACAGACCTGAT
AAATGTCTTCCATCATGTCCCGAGTTTCCTTAATAAAGTACTTTCTTTCTTAA
AAAGGACATGTTCTTTTCGTTTTTGGAAATTGACATATATGTCAGGCTTGTG
ATTCTTCTTGAATGGCATAAAAATGGTATCAATCGGTAA
>*nto-MIR7972c*
ATGGAAGGAGAATCACAAGCCTGACAAATATGTCACTTTCCAAAACCGGA
CTTTTTAAGAAAGAGGAACTTTATTATTGCGGACGCGTATTTGTCAGATTTG
TAATTCTCCTTCTGT

>*nta-MIR7972a*
ATTTTCTTGTCAAGGAGCAGAAGTAGAATTACAAATCTGCCAAATAAGTTT
CGCGTTTCTTTTAATAAAGTTACTCTTTCTTAAAAAAGAGACATGTTCTTTC
CGTTTTTGGAAAGTGACATATTTGTCAGGCTTGTGATTCTCCTTCCATGGCA
TAGAAAAT
>*nta-MIR7972b*
GAAGGAGAATTACAAATCTGGCAAATACGCGTCCGCAATAATAAAGTTCCT
CTTTCTTAAAAAGTCCGGTTTTGGAAAGTGACATATTTGTCAGGCTTGTGAT
TCTCCTTC
>*nta-MIR7972c*
TAAGGAGAATTACAGACCTGATAAATGTCTTCCATCATGTCCCGAGTTTCCT
TAATAAAGTACTTTCTTTCTTAAAAAGGACATGTTCTTTTCGTTTTTGGAAA
TTGACATATATGTCAGGCTTGTGATTCTTCTTG
>*nta-MIR7972d*
GAAGGAGAATTACAAAGGACAAATAAGTTTTCGGTCACGGGTTTCTTTTAA
TAAAGTTCCTCTTTCTTAAAAAGAGACATGTTCTTTCCGATTTTGGAAAGTG
ACATATTTGTCAGGCTTGTGATTCTCCTTC
>*nta-MIR7972e*
GAAGGAGAATTACAAATCTGACAAATAGGTTTCGGCGTCCCGGATTTCCTT
AATAAAGTTCTTTTTTCTTAAAAAAGACATGTTCTTTCCGTTTTTGGAAAGT
GACATATTTGTCAGGCTTGTGATTCTCCTTC
>*nta-MIR7972f*
TGATATAATTTTCTTGTTATGTAGAAGGAGAATTATAAGTCTTGCAATTAGGT
TTCGGTATCGCGGGTTTCCTTTAATAAATTTCTTCTTTCTTAAAAAAGACATG
TTCTTTCCTTCTTGGTTCCGTTTTTGGAAAGTGACATATTTGTCAGGCTTGT
GATTCTCCTTCCATGACACAGAAAATGGTACCA
>*nta-MIR7972g*
GTCGAAGGAGAATTACAAATCTAACAAATACACCAATTTCCTATAATAAAGT
TCTTCTTTCTTAGAAAGAGACATGTTCTTTCCGTTTTTGGAAAGTGACATAT
TTGTCAGGCTTGTGATTCTCCTTTGAT
>*nta-MIR7972h*
TTTCTTGTTATATTATAAGGAGAATTACAGACCTGATAAATATGTCTTCCATC
ATGTCCCGAGTTTCCTATAATAAAGTACTTCTTTCTTAAAAAGGGCATGTTC
TTTTCGTTTTTTGGAAATTGACATATTTGTCAGGCTTGTGATTCTTCTTGAAT
GGCATAAGAAA
>*nta-MIR7972i*
GAAGGAGAATTACAAATCTGACAAATAGACTTTCGTCCATTTCTTTGCCTTT
AATAAAGTTCTTCTTTCTTAGGAAGAGACGTTCTTTCCGTTTTTGGTAAGTG
ACATATTTGTCAGGCTTGTGATTCTCCTTT
>*nbe-MIR7972a*
GAGGAGAATCACAAGCCTGACAAATGTGTCTTTATCAAAAAAGCGATAATA
CATATTTTTCACATACAGGGCATTCTTTTGTAAATGAAATGGCAAAAATTATC
TAAACACCAGGAAATACTGTATTCTTTCTTTTAGAGATGGTCACACATATTT
GTCAGGTTTGTGATTCTCCTT

>*nbe-MIR7972b*
AGGAGAATCACAAACCTGACAAATATGTGTGACCATCTCTAAAAGAAAGA
ATACAGTATTTCCTGGTGTTTAGACAAATTTTTGTCATTTCATTTATAAAAGA
ATGCCCTGTTTGTGAAAAAAATGTCATATCGCGTTCTTGAAAAAGACATTTG
TCAAGCTTGTGATTTTCCT
>*nbe-MIR7972c*
AGGAAATCACAAACCTGACAAATATCTCTGACCATCTCTAAAAGAAAGAAT
ACATTATTTCCTGGTGTTTAGATAAATTTTTGTCATTTCATTTACAAAAGAAT
GCCCTATTTGTGAAAAAAATGTCATATCGCGTTCTTGAAAAAGACATTTGTC
AAGCTTGTGATTTTCCT
>*apa-MIR7972*
GTAACTATTTCTTGCCGAATCAAAGGAGAATTTCAAGCTTGATTAATAATTT
GAATTAGGATGAATCGGTGGGTCGCGGGCTGAGTGTTATTCGTCAGGCTTG
CCATTCTCCTTTGATGGCGTGGAATGGTCGC
>*jsa-MIR7972*
TTCGTGTCAAAATCAAAGTAGAATAACAAGCTTGACATACGAGTCGGCCGG
CGCTTGATTTCAAGGATACAGGCAGTAGTGCTAGTCATCGTGTTATATCATA
AAGATAAAGAGTTATTACAACAAAGAAAGCACTATCCATAAGGGCAGACTC
GTTTGTCAGGCTTGTCATTCTCCTTTGACGGCATCGAA
>*sob-MIR7972*
TTTGATGTTAAAATGATGGAGAATTACAAGCTTGATAAACGAGTTTTCCTCG
GCAGCTTAATCCGGTTCTAAACTATATGGTGCCAACACAGTAGTGGTGCTTC
AAACCCAACTAGCTAGCACTATATATAAAAAGGTAAGACTCTTTTGTCAGGC
TTGTCATTCTCCTTTGTTGGCATCGAA
>*fpe-MIR7972*
CAATGGAGAATTACAAGTTTGACAAATGAGCGTTTCTCGCGTCCTAAACTA
GTTCTAAACTACAGGGCGCACAGTAGTGCTAGATACCTAACTAGCTCTATTT
ATAAAAGGGATGCTCTTTTGTCAGGCTTGTCATTCTCCTTTG
>*oeu-MIR7972*
TGAGGGAGAATTACGAGCTTGACAAACGAGTTTAACTCGGCTGCTCCGATT
CCAACGACAGGGCACCCACCCAGTAGTGCTACAACCCCAACTAGCACTATT
TATAAAAGGTGGACTCTTTTGTCAGGCTTGTCATTCTCCTTTG
>*ofr-MIR7972*
TTCGATGTCGAATAAGGGAGAATTATGAGCTTGACAAACGAGTATTCCTCC
GCAGCTCATTCCGATTTTAAACTACATGGCGCCTATGCAGACTAGTGCTCCA
CGCACAACTAGCACTATTTATAAAAGGTGGACTCTTTTGTCAGGCTTGTCAT
TCTCCTTTGTTGGCATCGGA
>*avo-MIR7972*
CGAAGGAGAATTTCAAGCTTGACAAATACGTCTTTAATCAGTGAGTTTGAT
TCCACTTAATGGGAAAGGGTGCTGAAAAATGCAAGGTTCAAACTAAATTAA
GGAATCAGCTCTCTTTCCATTCTTGTTAAGTTTGGGGAAGACTTATTTGTCA
GGCTTGTGATTCTCCTTTG
>*rgl-MIR7972*
CTGAATCGAAGGAAAATTTCAAGCTTGACAAATACGTCTTCCATGGCGTCC

GTCCCAAACCAGTCTTCCTATATGTGGAAAGGGTACACAAAGGAAGAAAG
GATACACAAAAGACTAGCAAAATAGTCCACAATACTCTTTCCACCATTTTTA
AGGGAGTCAGTCTTATTTGTCAGGCTTGTTATTCTCCTTCGATGGCG
>*pja-MIR7972*
TTTCACGCCCAATTAAAGGAGAATTCCAAGCCCGACAAATACATCTTTCAT
GGCGAAAGAGTACTCAAAGAGACTTTAATTAAATAGTCAACAATACTCTTT
CCTCTTTAGGAAAGAGGAGACGAGTCTTATTTGTCAGGCTTGTGATTCTCC
TTTGATAGCGTGAAA
>*pke-MIR7972*
TCTTTCACGCCCAATTAAAGTAGAATCCCAAGCTCGACAAATGGCCTTAATA
TTAAAATAGTCAACAATTAAAAACTAGAACATATGTCTTATTTGTCAGGCTT
GTGATTCTCCTTTGATGGTATGAAATGA
>*cam-MIR7972*
CACACCGAATTAAAGGAGAATTTCAAGCTTGACAAATACGTCTTTCATGGC
GGCGGCCGTTCGAAACCAGTCTCAATTTATTTGGAAAGAGTACACAAAAA
GACCAAGTAAAATAGTCAACAATACTCTTTCCACTATTTCCAAGGGGACGA
GTCTTATTTGTCAGGCTTGTTATTCTCCTTTGATGGCGTG
>*pfo-MIR7972*
GTCGAAGGAGAATTTCAAGCTTAACAAATACGTCTTTGATGGCGTCCGTCC
GAAACCAACCTTTCTTTTCGCACAAAGACTACACAATAGACTAGTAAAATA
GTCAACAGTGATCTTTCCTATTTTTAGGAGGTGAGTCTTATTTGTCAGGCTT
GTCATTCTCCTTTGAC
>*pto-MIR7972*
TTTCACACTGAATCGAAGGAGAATTTCAAGCTTGACAAATACGTCTTTCAT
GGCGTCCGTCCGATACCAACCTTCCTTTTCGCATAAAGACTACACAATAGA
CTAGTAAAATAGTCAACAGTGATCTTTCCTATTTTTAGGAGGTGAGTCTTAT
TTGTCAGGCTTGTCATTCTCCTTTGACGGCGTGAAA
>*pov-MIR7972*
AATCGAGTGAGAATTTCAAGTCTGACAACTATAATGCATACGGTTAAACCA
CCTTTTGTTCATATTTGCCAGGCTTGTTATTCTCCTTTGATT
>*pla-MIR7972*
ATTGAAGTAGAATTTCAAGCTTGACAAATACGTCGTCCATGGCGTCCGAGA
GTAAGAACAGAACACATCCATATCTTCCTTACAATATGTGAAAGTGGGGA
CAGCTTACTACTAAAATAGTTAGCTTATCCCACTTTCACAAATTTGAGGAAT
TTGTTCGTATTTGTCAGGCTTGTTATTCTCCTCTGAT
>*ldu-MIR7972*
ATCGAGGGAGAATTTCAAGCTTGACAAATACGTCTTTCATGGCGTCCGTCC
GAAAAACTGGTATTTGGGAGAAAAGGAAAGAGAGCTAGTTGGTTAAAACT
AGAAAGACTAGTAAAATAGTCAATTCTACCCTCTTTTGTTCCCCACTTGGGC
CATTTTCTTTGGAGTCTTATTTGTCAGGCTTGTCATTCTCCTTTGAT
>*tgr-MIR7972*
TTTCAGGCTGAATCGAGGGAGAATTTCAAGCTTGACAAATACGTCTTTCAT
GGCGTCTGTCCGAAACCAGTCTTCCTTAATGTGGAAAGTGTACACGAAGG
ACTACTAAAATAGTCAACAGTGCTCTTTCCCCCTATTTTCTTTTCCCCTATTT

TCTAAGGAGGCGGGTCTTATTTGTCAGGCTTGTTATTCTCCTTTGATGGCGT
GAAA
>*oba-MIR7972*
ATCGAATGAGAATTTCAAGCTTGACAAATATGTCGGACTTGCCATCGTTCCG
AAAGCAGACTTCCTTAAAGAGTTTGAGAAGGAATTAACAGAAAGTACTCT
CTTTCTGCCATTTCTCTCTCTTGAGGAAGCAGTCTTATTTGTCAGGCTTGTTA
TTCTCCTTTGAT
>*pfr-MIR7972*
ATCGAGGGAGAATTTCAAGCTTGACAAATACGTTTGTGATGGCTTTCCTTAC
CGAAACCAGTCCTTTTAAGAGGAAAGGGCACATACAAGAAGGATTAAGTA
AATATAGTCAACACTACTCTTTCTCTCTCCCTCTCTCTCTCCCCCCTTTTCTC
TTAAAGGAAGCACTCTTATTTGTCAGGCTTGTTATTCTCCTTTGAT
>*rof-MIR7972*
TTTCAAGCTAAATCGAAGGAGAATTTCAAGCTTGACAAATACGTCTTTCATA
GCGTGAATCCGTGATCAGTCTTCTTTAATGTGGAAAGGGGTAAAGAAAAGG
GACTTGTAGTGAAAATAGTACTAATCCCTCTCTCTCACCACTTTTCTCTCTC
CCCTATAGAAGTAAGAAGTCTTATTTGTCAGGCTTGTTATTCTCCTACGATG
GCGTGAAA
>*msp-MIR7972*
GTCTATATCTTCGTCTGATACTTTTTCAGGTCGAATCGAAGGAGAATTTCAA
GCTTGACAAATACGTCTGATGGCGGCCATGCGGAAACCAGTCTTCCTTAATA
TGGATAGGGGCAAACAAAAGGGGATTAGTCAAAATAGTCAACACTTCTCTC
TCTCTCTCTCTCTCTCTCTCTCGTTTGCCCGTTTCTCTCTTATAAGGAAGCAG
TCTTATTTGTCAGGCTTGTTATTCTTCTTTGATGGCGTGAAAATGGTAGGAG
GCGGCGATTGGAT
>*ler-MIR7972*
TCGTGTATATCGCAGTAGAATTACGAGTCTGACAAATATATTTTCCGCCGCTA
TACCTTGCCAAAAATCACATCCTCAATTCTTTCCAAAATCACATCCTCAAAC
ACAGTTATATAGAAACCCAAACAAAGTCCAAACATCATCTTGTTTCTTGATT
TTATTATTTCTTTCATTTATTTCCCTTTTCTTGATTATCCAGTTTGTCTTTGGCA
TGGTGATATTTGTCAGGGGCGTGATTCTCCTTTGGTGGCATGA
>*aeu-MIR7972*
TTTTATGTCTATCGCAGTAGAATTACTATTCTGACAAATATATTTTCCGTCGCT
ACACCTTGCCAAAGAATCCTCTGTTCTTTCCATAATCAAAATCCTCACTTCT
TAGTTCTTTCCAGAATCACCTCTCTTCTTTCCAAAATCAAATCCTCACTTCTT
TCCAGAATCACATCCTCAAACACAGTTATATAGAGAACCAAGCAATCGAAG
TTAGATATTGTTTCAACAAGAATAGATTTTTGTTATTTCTTCAATTTATTTCCC
TCTTCTTGATCTTTGGCACGGTGATATTTGTCAGGGGCGTGATTCTCCTCCG
GTCGCATAAAA
